# Transcriptome analysis reveals potential immune function-related regulatory genes/pathways of female Lubo goat submandibular glands at different developmental stages

**DOI:** 10.7717/peerj.9947

**Published:** 2020-10-07

**Authors:** Aili Wang, Tianle Chao, Zhibin Ji, Rong Xuan, Shuang Liu, Maosen Guo, Guizhi Wang, Jianmin Wang

**Affiliations:** Shandong Provincial Key Laboratory of Animal Biotechnology and Disease Control and Prevention, College of Animal Science and Veterinary Medicine, Shandong Agricultural University, Taian, P.R. China

**Keywords:** Transcriptome, Different ages, Goat, Submandibular gland, High-throughput sequencing

## Abstract

**Background:**

The submandibular glands, as major salivary glands, participate in rumen digestion in goats. Sialic acid, lysozyme, immunoglobulin A (IgA), lactoferrin and other biologically active substances secreted in the submandibular glands were reported in succession, which suggests that the submandibular gland may have immune functions in addition to participating in digestion. The aim of this study was to map the expression profile of differentially expressed genes (DEGs) at three different stages by transcriptome sequencing, screen immune-related genes and pathways by bioinformatics methods, and predict the immune function of submandibular glands at different developmental stages.

**Methods:**

Nine submandibular gland tissue samples were collected from groups of 1-month-old kids, 12-month-old adolescent goats and 24-month-old adult goats (3 samples from each group), and high-throughput transcriptome sequencing was conducted on these samples. The DEGs among the three stages were screened and analysed. Key genes and signalling pathways were selected via protein-protein interaction (PPI) network analysis.

**Results:**

The results revealed 2,706, 2,525 and 52 DEGs between 1-month-old and 12-month-old goats, between 1-month-old and 24-month-old goats, and between 12-month-old and 24-month-old goats, respectively. Gene Ontology (GO) and Kyoto Encyclopedia of Genes and Genomes (KEGG) analyses indicated that most of the DEGs were enriched in immune- related GO terms and pathways. Based on functional enrichment analysis and network analysis, 10 genes (*PTPRC, CD28, SELL, LCP2, MYC, LCK, ZAP70, ITGB2, SYK* and* CCR7*), two signalling pathways (the T cell receptor signalling pathway and the NF-κβ signalling pathway) and eight GO terms (T cell receptor signalling pathway, neutrophil mediated immunity, B cell mediated immunity, regulation of alpha-beta T cell activation, positive regulation of T cell proliferation, regulation of leukocyte differentiation, positive regulation of antigen receptor-mediated signalling pathway, positive regulation of lymphocyte proliferation) that may play key roles in the immune functions of the goat submandibular glands at different developmental stages were identified. Moreover, we found that eight antibacterial peptide-encoding genes were downregulated in the tuberculosis and salivary secretion pathways, while all immunoglobulins were upregulated in 10 immune system pathways. These findings indicate that the submandibular glands may be important immunological organs during the growth process of goats and that the immune function of these glands gradually weakens with age up to 12 months but remains relatively stable after 12 months of age. Overall, this study will improve our understanding of transcriptional regulation related to goat submandibular gland immune function.

## Introduction

The submandibular glands are the second-largest salivary glands in mammals. They can secrete many biologically active substances during development and are some of the most important glandular tissues in animals (Kaplan & Baum, 1993). The submandibular glands of mice and rats contain immunosuppressive factors with molecular weights (MWs) of 30,000 and 40,000 Daltons, respectively (Abdelhaleem & Sabbadini, 1992; Ikematsu, Pour & Kazakoff, 1997). Several antimicrobial substances, such as lysozyme, immunoglobulin A (IgA), lactoferrin and β-defensin, have been found in rat submandibular glands, especially in serous acini and ductal cells (Carpenter et al., 2004). IgA has been detected in the secretions of submandibular glands in humans, rats, and cattle (Carpenter et al., 2004; Sakaguchi et al., 2013). In addition, some studies have found erythroid differentiation factor, endothelin, hepatocyte growth factor (HGF), metastatic growth factor (MGF), transforming growth factor-α (TGF-α), and other factors in the submandibular glands (Amano et al., 1994; Ikematsu, Pour & Kazakoff, 1997). Other factors have revealed the presence of vasopressin, epidermal growth factor (EGF), nerve growth factor (NGF), kallikrein, MGF and HGF in the submandibular glands (He et al., 2011; Kurabuchi, Gresik & Hosoi, 1999; Miyaji, Aiyama & Kurabuchi, 2008). Several bioactive proteins, including kallikrein, calpain 3 and calcitonin, have been found in the submandibular glands of mice (Kumchantuek et al., 2016; Kurabuchi, Gresik & Hosoi, 1999; Zhang et al., 2014). Bioactive monoamine substances have also been discovered in rodent submandibular gland cells using immunohistochemistry. Notably, Mathison et al. (1992) found that the submandibular glands may play a key role in protection against pulmonary inflammation caused by decentralization of the superior cervical ganglia. Furthermore, high-MW sialoglycoproteins and hydrophobic peptides have been isolated from human and rat submandibular glands ( Reid & Heywood, 1988; Tabak et al., 1985). A previous study detected muscarinic receptor (M-receptor) subtypes in sheep submandibular glands by immunoblotting and immunohistochemistry (Ryberg et al., 2008).

The goat submandibular gland comprises serous acini and mucinous acini, with mucinous acini accounting for the majority. In addition to secreting saliva, which plays important roles in maintaining oral health and protecting gastrointestinal epithelial cells (Nagler, 2004), the submandibular gland also secretes large amounts of neutral glycoproteins and few acidic glycoproteins (Habata, Yasui & Tsukise, 2011; Ryberg et al., 2008; Sakaguchi et al., 2013). EGF and several antibacterial substances, such as lysozyme, IgA, lactoferrin, and β-defensin, which participate in the formation of the immune system, have been found in serous acinar and ductal cells (Abdelhaleem & Sabbadini, 1992; Habata et al., 2012). Histatin 5, a member of the group of low-MW salivary proteins that is secreted by the submandibular glands, inhibits the levels of bacterial and host-derived proteolytic enzymes in oral inflammatory exudates (Gusman et al., 2001). Thus, goat submandibular glands may be involved in immune defence and immunoregulatory functions (Habata, Yasui & Tsukise, 2011).

The function of the submandibular glands is related to age (Ghezzi & Ship, 2003; Jaskoll et al., 2001). Gene expression in submandibular glands differs greatly between young (8-week-old) and adult (50-week-old) mice (Saito et al., 2015). Before weaning, goats exhibit low chewing activity, a lack of secretory granules and surrounding inclusions, and low saliva secretion; after weaning, they exhibit enhanced chewing activity and secretion of large amounts of protein. The structures and secretory granules of goat parotid salivary glands also differ between milk-suckling kids and diet-fed goats (Elewa et al., 2014). The submandibular gland may have similar changes.

Thus far, studies on goat submandibular glands have mainly focused on the identification of cellular components and structures via immunohistochemistry; relevant data on mRNA expression levels in tissues at specific phases are lacking. Characterization of mRNA expression in goat submandibular glands at different developmental stages is helpful for understanding the role of the submandibular glands in goat development. Therefore, in this study, transcriptome analysis was performed on submandibular gland samples from 1-month-old kids, 12-month-old unbred adolescent goats, and 24-month-old adult goats to investigate DEGs expressed in these glands, with a focus on genes related to immune-related pathways. The key regulator genes involved in immunity might contribute to candidate genes for improvement of goat immunity.

## Materials and Methods

### Ethics statement

All animal experiments were approved by the Committee on the Management and Use of Laboratory Animals of Shandong Agricultural University (Permit Number: SDAUA-2018-052) and conducted in accordance with the “Guidelines for Experimental Animals” of the Ministry of Science and Technology (Beijing, China). Every effort was made to minimize pain.

### Sample collection and RNA extraction

The collection of experimental animals was carried out according to the principle of the 3Rs (Reduction, Replacement, and Refinement) for animal experimentation. Goats were separated into three different groups based on their developmental stages: the 1-month-old group, the 12-month-old group and the 24-month-old group. Three healthy female goats (Lubo goats) from each group (total, nine goats) were selected as experimental animals. The animals were obtained from Shandong Fengxiang Animal Husbandry and Seed Industry Technology Co., Ltd. (Laiwu District, Jinan, Shandong Province, China). All goats were raised under the same conditions with natural lighting and free access to food. All goats were fasted from food for 24 h and fasted from liquids for 2 h, and then xylazine hydrochloride (Huamu Animal Health Products Co., Ltd., China) was injected intramuscularly to induce general anaesthesia. After slaughter and exsanguination, the submandibular glands were immediately collected surgically, placed in liquid nitrogen and then placed in a −80 °C freezer for long-term storage. To distinguish the samples, the submandibular salivary gland samples from 1-month-old goats were labelled A1-L, A2-L and A3-L; the samples from 12-month-old goats were labelled B3-L, B4-L and B5-L; and the samples from 24-month-old goats were labelled C2-L, C3-L and C5-L. Total RNA was extracted from the 9 submandibular gland samples using TRIzol reagent (Invitrogen, Carlsbad, CA, USA). The integrity and quality of the extracted RNA was determined by measuring the absorbance at 260/280 nm (A260/A280) using an Agilent BioAnalyzer 2100 (Agilent, Shanghai, China). The short read alignment tool Bowtie2 (2.2.8) (Langmead & Salzberg, 2012) was used to map the reads to a ribosomal RNA (rRNA) database. The rRNA-mapped reads were then removed, and all other RNA reads were retained.

### Library construction

Total RNA was randomly cleaved into short fragments. To synthesize first-strand cDNA, the RNA fragments were used as templates in a reaction with random hexamers, buffered dNTPs (with dUTP instead of dTTP) and RNase H. Second-strand cDNA was synthesized using DNA polymerase I. The cDNA was purified using a QIAquick PCR kit (Qiagen) and eluted with EB buffer (10 mM Tris-HCl, pH 8.0). The sticky ends were repaired, and an adaptor sequence was ligated to the A base at the 3′ end of each cDNA molecule; the second strand was subsequently degraded with uracil-N-glycosylase (UNG). The ligated products were reverse transcribed by PCR amplification, and the products were purified to generate a cDNA library. After generation, the cDNA library was sequenced using an Illumina HiSeq™ 4000 from Gene Denovo Biotechnology Co. (Guangzhou, China).

### Alignment with reference genome

To obtain high-quality clean reads, all raw reads were further filtered by removing the reads containing adapters, reads that were less than 50 nt, reads containing poly N, and low-quality reads from raw data. Then, the alignment tool Bowtie2 (2.2.8) was used to map the high-quality clean reads to an rRNA database (mismatch number: 0), and the mapped rRNA reads were removed (Langmead & Salzberg, 2012). The comparison software TopHat2 (2.1.1) was used to align the remaining reads to the goat reference genome (http://www.ncbi.nlm.nih.gov/genome/?term=goat) (Kim et al., 2013). The TopHat2-based alignment results were used to reconstruct the transcripts using Cufflinks. Based on the positions of the assembled transcripts on the reference genome and the screening criteria of a transcript length ≥200 bp and a number of exons ≥2, known transcripts and new transcripts were obtained.

### Screening of differentially expressed genes (DEGs)

Transcript abundances were quantified by RSEM software (Li & Dewey, 2011). FPKM (Fragments Per Kilobase per Million) was used as the standard for screening differentially expressed transcripts. EdgeR v3.26.8 was used for differential mRNA expression analysis among the 3 age groups (Robinson, McCarthy & Smyth, 2010). To adjust the *P*-values, the Benjamini–Hochberg method for controlling the FDR was used (Guo & Bhaskara Rao, 2008). Genes with FPKM >1, false discovery rate (FDR) < 0.05 and a |log2(fold change)| > 2 were considered significantly differentially expressed (Trapnell et al., 2010). Differentially expressed coding RNAs were then subjected to enrichment analysis of GO functions and KEGG pathways (Kanehisa & Goto, 2000).

Based on the expression levels of the transcripts, the relationships between samples and transcripts were hierarchically clustered, and heat maps were used to present the clustering results. The expression scale of 9 groups of samples was *Z*-Score standardized using the R language scale function, and the heat map was constructed using ggplot two packages in R (version 3.2).

### Gene Ontology (GO) and Kyoto Encyclopedia of Genes and Genomes (KEGG) pathway enrichment analyses

GO and KEGG pathway enrichment analyses were performed using the R package clusterProfiler. The DEGs were mapped to the GO database (http://www.geneontology.org/), and the obtained genes for each term were counted to obtain a list of transcripts enriched for specific GO terms.

The DEGs were mapped to the KEGG database (https://www.genome.jp/kegg/) for KEGG pathway analysis using the same method as that used for the GO analysis. In the GO and KEGG pathway analyses, the FDR after *p* value calibration was used as the screening standard, and pathways with FDR values ≤0.05 were considered significantly enriched pathways.

### Protein–protein interaction (PPI) network analysis and gene immune function network construction

Based on information from STRING v.10.0, a PPI network was constructed for the protein-coding genes (Szklarczyk et al., 2015). Then, credible interactions (with combined scores ≥ 0.4) were accepted for further network analysis. Cytoscape (version 3.5.1) was also used (Shannon et al., 2003). The two resulting networks were merged to obtain the final interaction network. The degree-sorted layout was used for interaction degree analysis (in which all nodes were sorted by their interaction degree values).

The gene immune function network was constructed using the R Package ‘enrichplot’ (https://github.com/YuLab-SMU/enrichplot) with 18 genes and eight GO terms selected from the PPI network and GO/KEGG analyses.

### Verification using real-time quantitative PCR (RT-qPCR)

A total of 18 DEGs were randomly screened from the transcriptome sequencing results for RT-qPCR analysis. The RT-qPCR primers used for validation were designed according to the sequences of selected DEGs using Primer Premier 5.0 (Premier, Canada), which are provided in Table 1. cDNA was synthesized by using the Primerscript RT reagent Kit (TaKaRa) with 1 µg of total RNA. RT-qPCR was performed using SYBR Premix Ex Taq II (TaKaRa, DRR081A) and conducted using a Stratagene Mx3000 real-time system (USA). A reaction volume of 15 µL with 0.3 µL of forwarding primer (0.4 mmol each), 0.3 µL of reverse primer (0.4 mmol each), 7.5 µL of 2X SYBR Premix Ex Taq II reaction buffer, 0.3 mL of 50X ROX Reference Dye II, 1.5 µL of cDNA (10 pg/mL, 1 mg/mL), and 5.1 µL of RNasE-free dH_2_O was used. The reaction mixtures were incubated in a 96-well plate in an Mx3000p SYBR Green real-time quantitative PCR analyser (Stratagene, USA) at 95 °C for 30 s, followed by 40 cycles of 95 °C for 5 s and 60 °C for 30 s. All reactions were performed in triplicate. ACTB was used as a reference gene. After amplification, the 2^−ΔΔ*CT*^ method was used to calculate gene expression levels, the significance of gene expression between different groups was tested by Tukey’s HSD, and gene expression histograms between different groups were performed by GraphPad software.

**Table 1:** Primer sequences.

**Transcript**	**Gene**	**F**		**R**		
**NM_001314142.1**	TGFB1	CTGAGCCAGAGGCGGACTAC		TGCCGTATTCCACCATTAGCA		
**XM_005676186.2**	CXCR4	CGCTCGAGTCTGTCTCAACCGAAT		ATGCGGCCGCTAGGTTTATCATGT		
**XM_018065503.1**	PTGER4	GTGGTGCTCTGTAAGTCGC		GATGAAGGTGCTGTACTCGCA		
**XM_018064894.1**	COL1A1	TCCAACGAGATCGAGATCC		AAGCCGAATTCCTGGTCT		
**NM_001314219.1**	IGFBP3	AAAGGTCATGCCAAGGACAG		TGCCCGTACTTATCCACACA		
**XM_005676403.3**	CD28	GGAGGTCTGTGCTGTGAATGG		CGGTGCAGTTGAATTCCTTATTT		
**NM_001314325.1**	FOXM1	TGCCCACCCTCTGATATGGA		TGCTGAGGCTGTCGTTCATT		
**XM_005695059.3**	BCL2A1	ACTGCCAGAACAATATTCAACC		GTAAGAATACCTTCAAAGGCGA		
**XM_005696606.3**	TNF	TCGTATGCCAATGCCCTCA		GATGAGGTAAAGCCCGTCAGT		
**XM_005693201.3**	CCL5	CCATGGCAGCAGTTGTCTTT		CACCCACTTCTTCTCTGGGT		
**XM_018056969.1**	GATA3	CCGTGGTGTCTGTGTTCTCACT		TCAATAGGGAATGTGAGTCTGAATG		
**XM_018058563.1**	MYC	GTGGTCTTCCCCTACCCGCTCAACGA		ATCTCTTTAGGACCAACGGGCTGTGA		
**XM_005681326.3**	LEF1	CCCCACCTCTTGGCTGGTTTTCTCA		TTGGCTCCTGCTCCTTTCTCTGTTC		
**XM_005686292.3**	IL1R2	CGCCAGGCATACTCAGAAA		GAGAACGTGGCAGCTTCTTT		
**XM_018055547.1**	DNMT3a		AGCACAA CGGAGAAGCC		TTCCAGGAAGCAGTTCTTG	
**XM_018062796.1**	IRF3		GAGGACCACAGCAAGGACTC		TGTCTGCCATTGTCTTGAGC	
**XM_005690795.3**	ISG15		CAGTTCATCGCCCAGAAGAT		GTCGTTCCTCACCAGGATGT	
**XM_018051135.1**	INSR		ATGCCCTGGTGTCACTTTCCTTCT		TTAGGTTCTGGTTGTCCAAGGCGT	

## Results

### Summary of sequencing data

We constructed nine submandibular gland cDNA libraries for Lubo goats of three ages. A total of 862,995,872 raw reads were obtained from sequencing. After screening, 853,786,462 clean reads were obtained, accounting for more than 98% of the total number of reads (Table S1). The alignment rates for the nine libraries with the goat genome were above 80%; specifically, the mapping rates for A1-L, A2-L, A3-L, B3-L, B4-L, B5-L, C2-L, C3-L and C5-L were 91.02%, 93.13%, 91.66%, 88.54%, 87.30%, 88.00%, 83.76%, 84.49% and 87.57%, respectively (Table S2). All data have been uploaded to the Gene Expression Omnibus (GEO) database under the accession number GSE144368.

The numbers of known and new mRNAs that were identified and predicted in each library are provided in Table S6. As shown in Table 2, a total of 42,686 mRNAs were detected in all libraries: 41,914 in the 1-month-old goat library (group A), 39,070 in the 12-month-old goat library (group B), and 37,923 in the 24-month-old goat library (group C). Overall, 35,451 known mRNAs were detected in all libraries, accounting for 83.05% of the total number of mRNAs. The numbers of known mRNAs in group A, group B and group C were 32,875 (77.02% of the total), 30,577 (71.63% of the total), and 29,634 (69.42% of the total), respectively. A total of 10,053 new mRNAs were found in all libraries, including 9,039 in group A, 8,493 in group B, and 8,289 in group C (Table 2). The C5-L library had the lowest numbers of known mRNAs, new reference mRNAs, and total mRNAs (23,946, 6,080, and 30,026, respectively). The A3-L library had the most known mRNAs, new mRNAs, and total mRNAs (28,581, 7,549, and 36,130, respectively).

**Table 2:** Statistics of known mRNAs and new transcripts of different age groups. A were the samples for goats of 1-month-old (group A), B were the samples for goats of 12- month-old (group B), C were the samples for goats of 24-month-old (group C).

**Group Name**	**All Reference mRNAs**	**Known mRNANum**	**New mRNANum**
**A**	41914	32875 (77.02%)	9039
**B**	39070	30577 (71.63%)	8493
**C**	37923	29634 (69.42%)	8289
**Summary**	42686	35451 (83.05% )	10053

### Identification and analysis of DEGs

**Figure 1:**
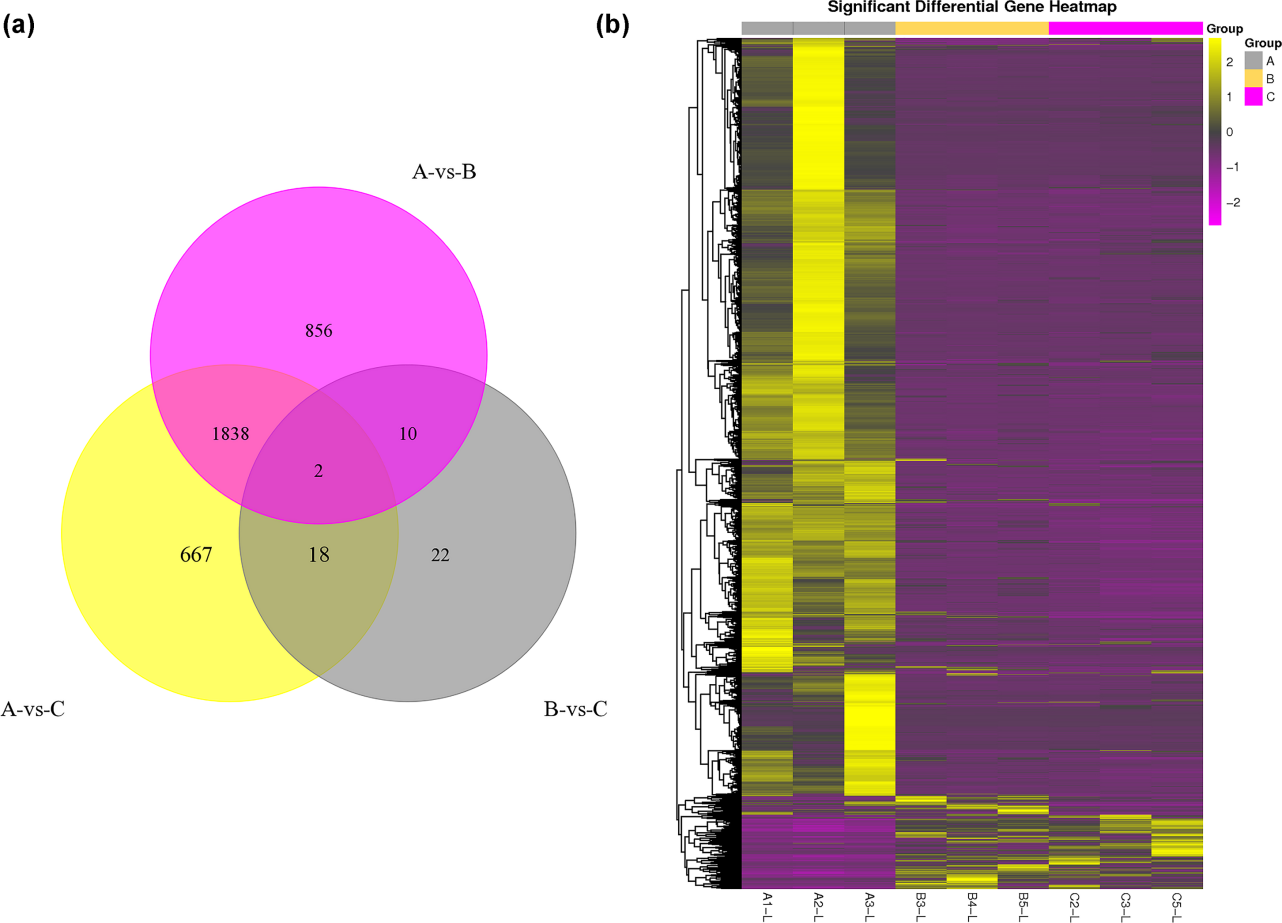
Statistical summary and clustering pattern of DEGs between three different age groups. (A) Statistical summary of DEGs between three different age groups. A were the samples for goats of 1-month-old (group A), B were the samples for goats of 12-month-old (group B ), C were the samples for goats of 24-month-old (group C). (B) Clustering pattern of DEGs between groups. Each column in thefigure represents a sample, and each row represents a transcript. The expression of transcripts in different samples was indicated by different colors. The yellower the color, the higher the expression, and the deeper the purple, the lower the expression.

The DEG analysis revealed 2,706 and 2,525 differentially expressed mRNAs between group A and group B and between group A and group C, respectively, but only 52 differentially expressed mRNAs between group B and group C. These results may indicate that mRNA expression levels in the submandibular salivary glands of 12-month-old goats and 24-month-old goats are highly similar. Numerous mRNAs were downregulated in group B and group C compared with group A (Fig. 1A). The clustering diagram of the differential expression patterns between the groups (Fig. 1B) shows that there were many DEGs between group A and group B and between group A and group C, while there were no significant DEGs between group B and group C.

### GO and KEGG pathway analyses

**Figure 2:**
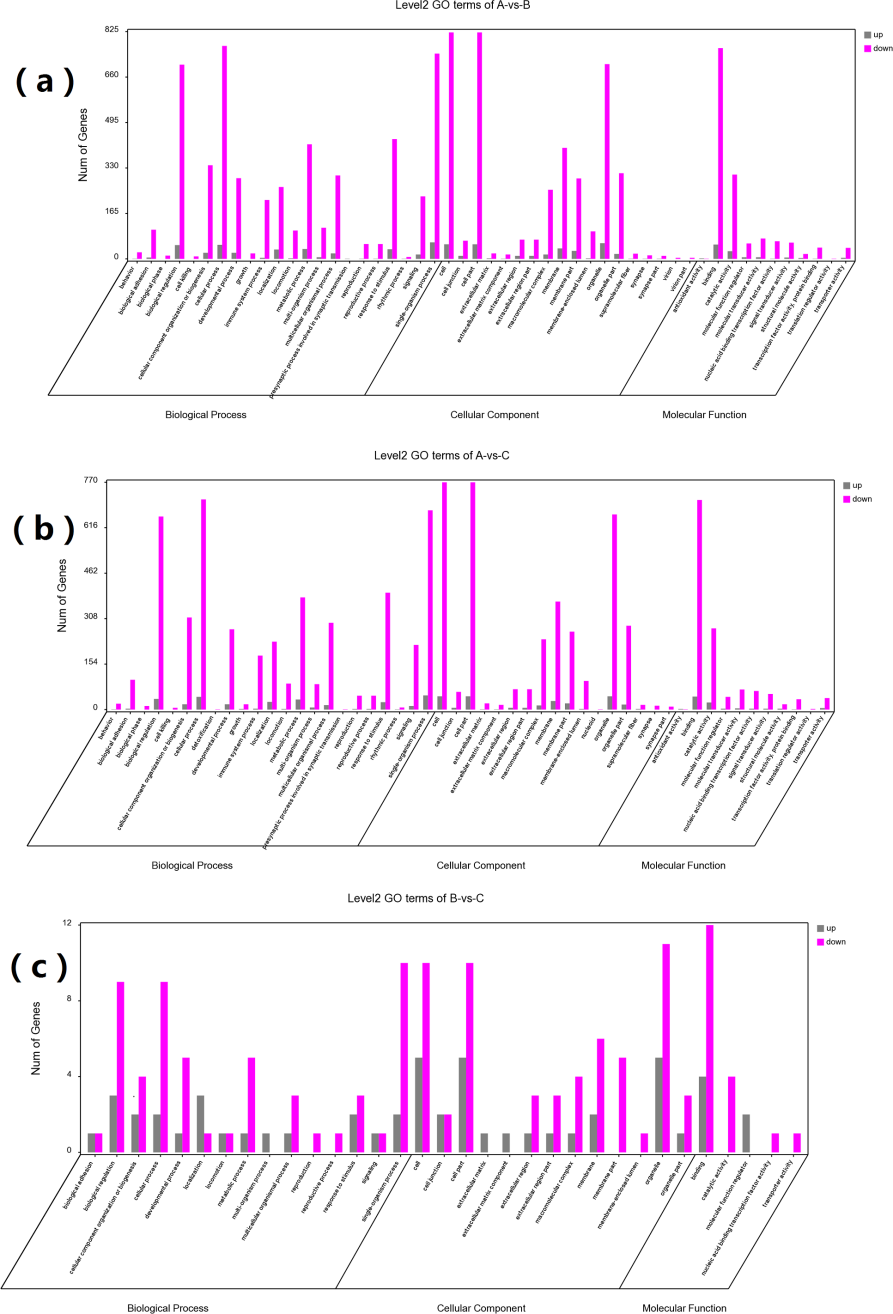
GO analysis of DEGs between different groups. The DEGs are classified into three categories: cellular component, molecular function, and biological process. The percentage of genes in each category and the number of genes are shown above. (A) GO analysis of DEGs between groups A and B. (B) GO analysis of DEGs between groups A and C. (C) GO analysis of DEGs between groups B and C.

**Figure 3:**
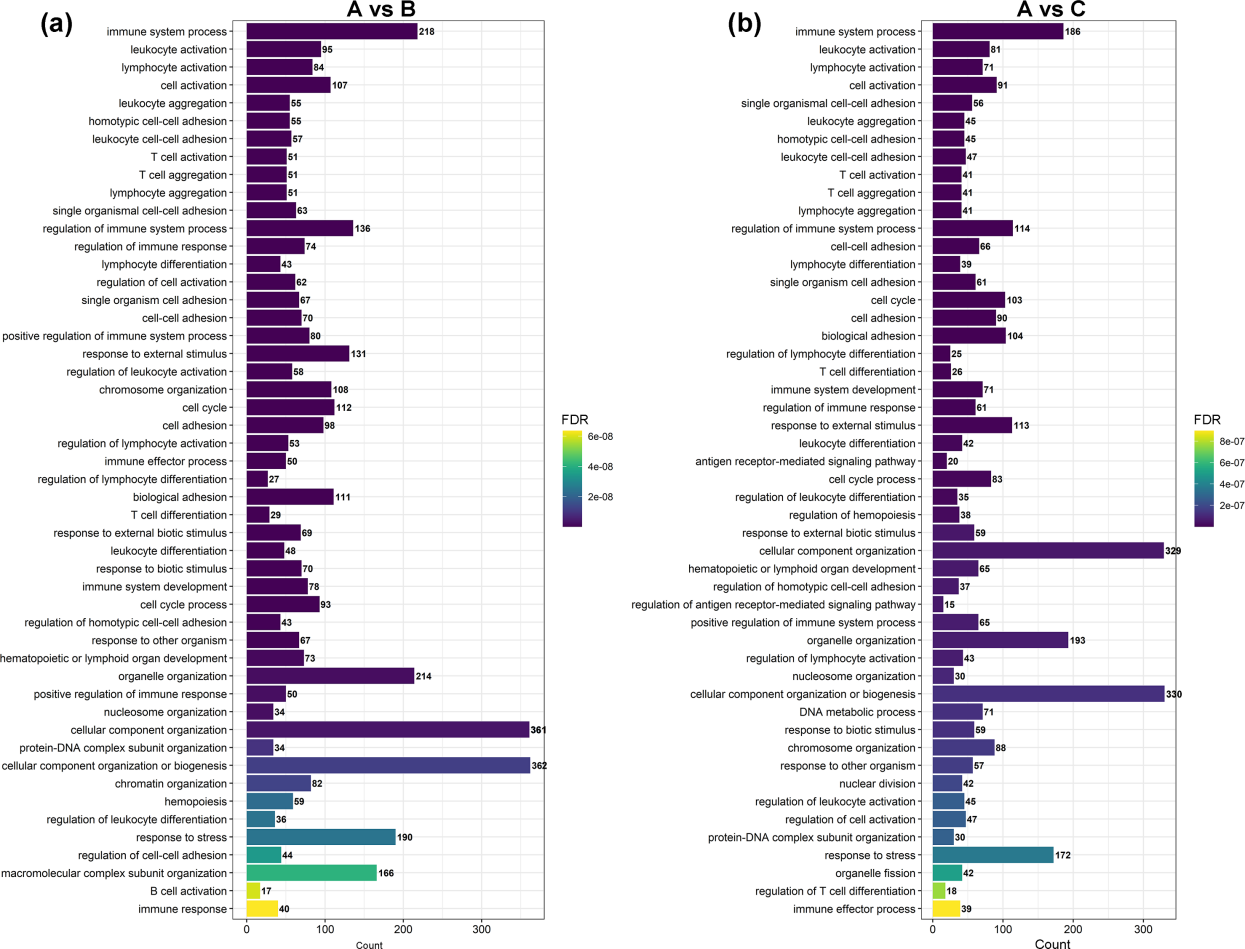
The most enriched GO terms of group A vs. B and group A vs. C in biological process. (A) The most enriched GO terms of group A vs. B. (B) The most enriched GO terms of group A vs. C.

GO and KEGG enrichment analyses were performed on DEGs to further confirm their involvement in the regulation of biological processes. The GO analysis results are shown in Figs. 2 and 3. In the DEG enrichment analysis between group A and group B, 3,673 GO terms were obtained, among which 427 GO terms were significantly enriched, including 46, 16 and 365 GO terms in the cellular component, molecular function and biological process categories, respectively (FDR < 0.05) (Fig. 2A; Table S7). Between group A and group C, 3,569 GO terms were obtained, among which 362 GO terms were significantly enriched, including 41, 21, and 300 GO terms in the cellular component, molecular function and biological process categories, respectively (FDR < 0.05) (Fig. 2B; Table S8). Between group B and group C, 516 GO terms were obtained, but there were no significantly enriched annotations (FDR < 0.05) (Fig. 2C). Notably, the top four significantly enriched terms for the DEGs between group A and group B and the DEGs between group A and group C were the same; these terms were the immune system process (GO:00023761, FDR = 6.26E−38 and 1.44E−27, respectively), leukocyte activation (GO:0045321, FDR = 5.44E−33 and 2.78E−25, respectively), lymphocyte activation (GO:0046649, FDR = 9.28E−32 and 5.51E−24, respectively) and cell activation (GO:0001775, FDR = 2.57E−31 and 1.16E−23, respectively) terms. At the same time, most of the significantly enriched GO terms of group A vs. B and A vs. C in Biological Process were immune-related terms (Fig. 3).

**Figure 4:**
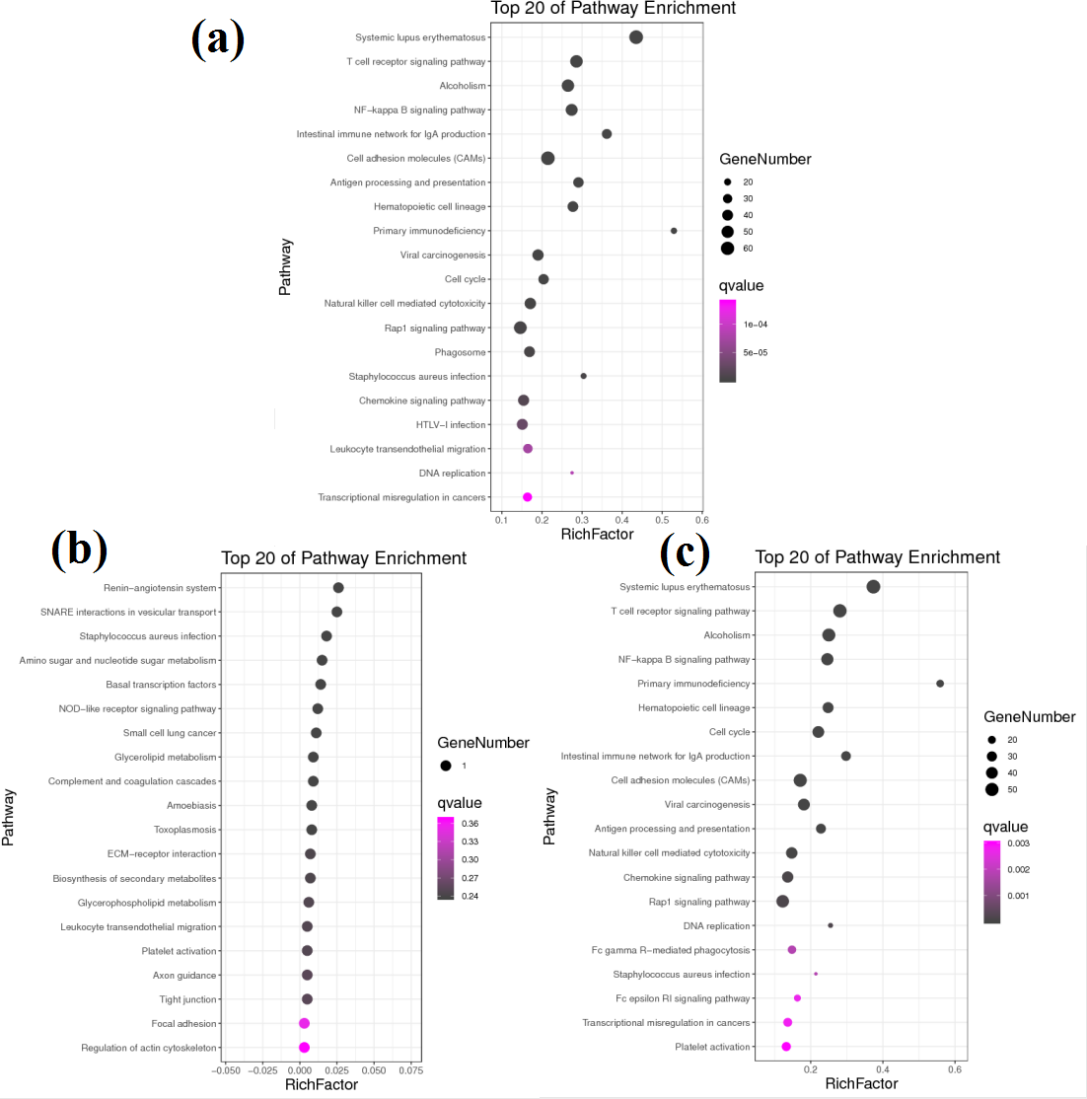
KEGG pathway analysis of DEGs between different groups. (A) KEGG pathway involved by DEGs between groups A and B. (B) KEGG pathway involved by DEGs between groups B and C. (C) KEGG pathway involved by DEGs between groups A and C.

KEGG pathway analysis helped to further elucidate the biological functions of the transcripts (Fig. 4). Analysis of the differentially expressed mRNAs among goats at 3 developmental stages revealed 769 DEGs between group A and group B, of which 456 DEGs were significantly enriched in 47 KEGG pathways (FDR < 0.05). Among all significantly enriched pathways, the systemic lupus erythaematosus (FDR = 1.90E−33) and T cell receptor (TCR) signalling pathways (FDR = 1.14E−17) were the most highly enriched (Fig. 4A). A total of 683 DEGs were identified between group A and group C, of which 390 DEGs were significantly enriched in 31 KEGG pathways (FDR < 0.05) (Fig. 4B). The pathways with the highest degrees of enrichment were again the systemic lupus erythaematosus (FDR = 3.97E−27) and TCR signalling pathways (FDR = 3.09E−19). A total of 11 DEGs were identified between group B and group C that participated in 23 signalling pathways (Fig. 4C). However, only one gene was enriched per pathway, and no pathways reached a level of significant enrichment.

Notably, more than half of all significantly enriched KEGG pathways between groups A and B and between groups A and C were related to immunity. Among the 47 significantly enriched KEGG pathways between groups A and B, 35 pathways belonged to the immune system and immune disease classes, and 12 other pathways belonged to the cancer and infectious disease classes. Similarly, among the 31 significantly enriched KEGG pathways between groups A and C, 20 pathways belonged to the immune system and immune disease classes, and 11 other pathways belonged to the cancer and infectious disease classes. These results reveal that there are transcription level differences in the submandibular glands between 1-month-old kids and adult goats. The DEGs between groups A and B and between groups A and C were all enriched in 10 identical immune system pathways. However, the Q values were different, and the upregulated genes were not exactly the same in both comparisons. The immunoglobulins involved in these 10 pathways were upregulated, while almost all the other genes were downregulated. The Q values and upregulation levels of the DEGs in the 10 immune system pathways between groups A and B and between groups A and C are shown in Table 3.

**Figure 5:**
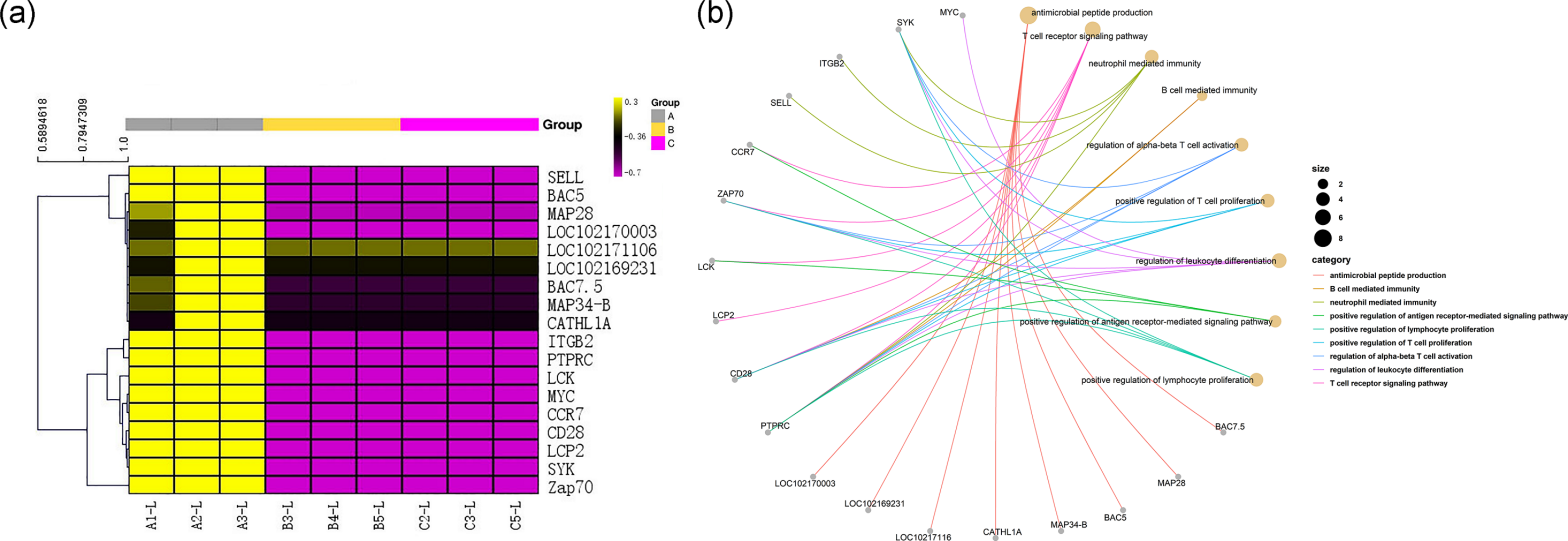
Gene immune function network analysis. (A) Heat map of expression quantity of eight antimicrobial peptides in nine samples. (B) Gene immune function network analysis of 10 genes selected by PPI analysis and the eight antimicrobial peptides.

Among the DEGs, genes encoding 8 antibacterial peptides, including the BAC7.5 protein precursor, MAP28, BAC5, MAP34-B, CATHL1A, LOC102171106, LOC102169231 and LOC102170003, were relatively highly expressed in the transcriptome of group A (FPKM values: 162.88, 50.45, 46.60, 43.32, 22.67, 35.32333, 29.97333, and 123.5833, respectively). However, there was almost no expression of the above 8 peptide-encoding mRNAs in group B (FPKM values: all 0.001) and in group C (FPKM values: 0.001, 0.001, 0.026667, 0, 0.001, 0.001, 0.001, and 0.001, respectively). The above results are shown in Fig. 5A.

**Table 3:** Up-regulated genes in 10 immune system pathways between groups A and B, groups A and C. A were the samples for goats of 1-month-old (group A), B were the samples for goats of 12- month-old (group B ), C were the samples for goats of 24-month-old (group C).

**Pathway**	**A-vs-B**		**A-vs-C**	
	**up-regulated genes**	**FDR**	**up-regulated genes**	**FDR**
T cell receptor signaling pathway	no	1.14E−17	no	3.09E−19
Intestinal immune network for IgA production	BCR IgA	7.90E−15	BCR IgA	3.09E−11
Antigen processing and presentation	HSP70	7.42E−13	no	1.12E−08
Hematopoietic cell lineage	IgM,IgD	8.40E−13	IgM,IgD	2.36E−11
Natural killer cell mediated cytotoxicity	IgG	4.13E−07	IgG	8.75E−06
Chemokine signaling pathway	no	1.11E−05	no	8.03E−05
Leukocyte transendothelial migration	RhoGAP	7.35E−05	no	2.53E−02
Fc gamma R-mediated phagocytosis	IgG	3.47E−04	IgG	1.76E−03
Fc epsilon RI signaling pathway	PKC ,IgE	1.47E−02	PKC ,IgE	2.69E−03
Platelet activation	p190	3.51E−02	no	2.96E−03

### Screening of key regulatory genes

To further identify the key regulatory genes that caused the differential enrichment of salivary gland immune-related pathways between the different age groups, a PPI network analysis was performed. According to the KEGG pathway enrichment results, a total of 27 significantly enriched pathways overlapped between the two comparisons (A vs. B and A vs. C); these pathways contained 290 genes that overlapped between both comparisons.

**Figure 6:**
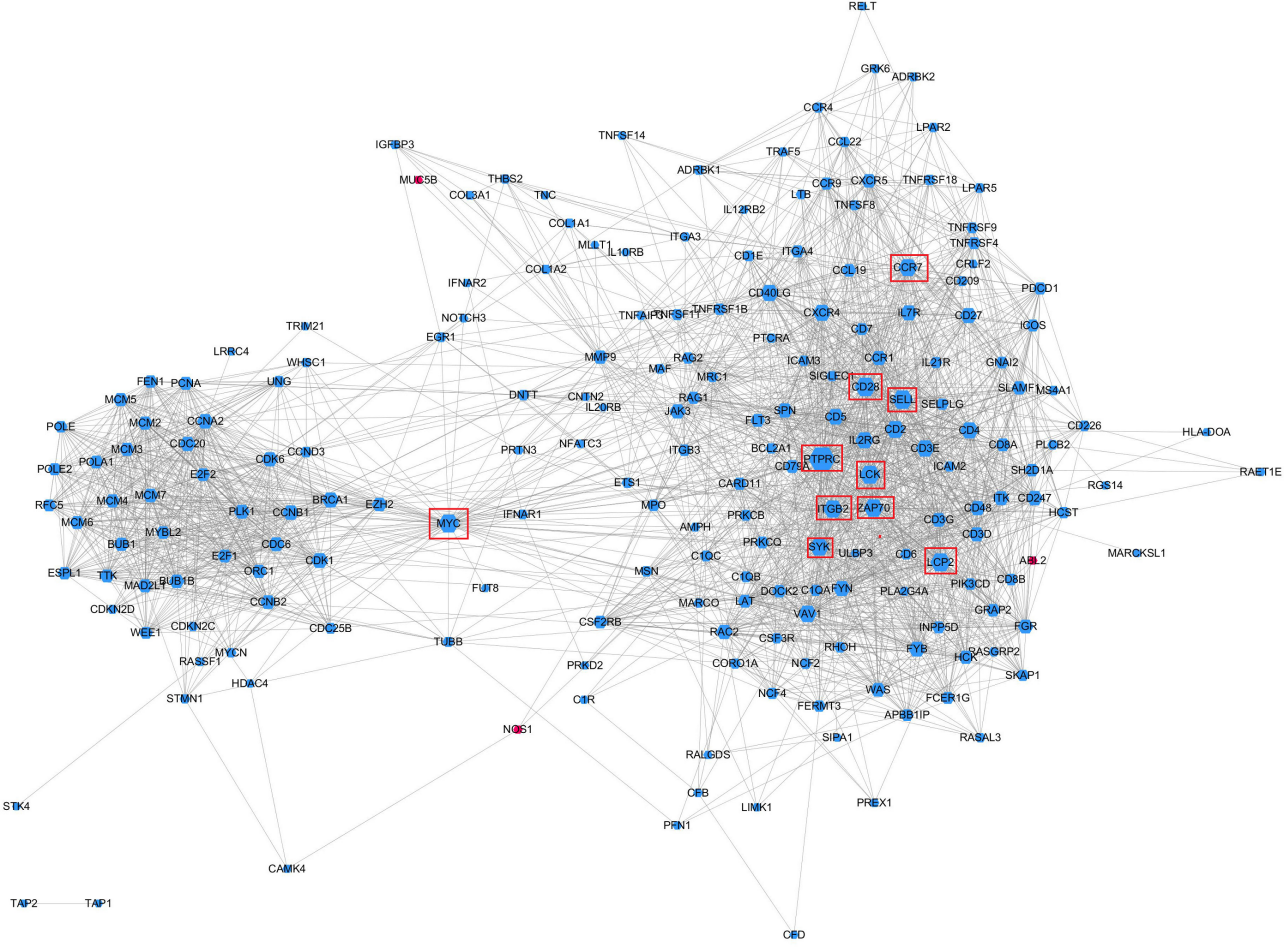
Protein–protein interaction networks of KEGG pathway significantly enriched genes. Gene node size was decided by the interaction degree value. Red node, Higher expressed in groups B and C; Blue node: Higher expressed in group A; Red boxes, potential key regulatory genes.

All 290 genes were used for PPI network construction. A total of 2,319 interactions among 202 genes were obtained (Fig. 6). All genes in the network were ranked by interaction degree, and the top 10 were selected as potential key regulatory genes: PTPRC, CD28, SELL, LCP2, MYC, LCK, ZAP70, ITGB2, SYK and CCR7. We then performed KEGG enrichment analysis for the 10 key regulatory genes, and two signalling pathways were determined to be significantly enriched: the TCR signalling pathway (FDR = 5.0E−05) and the NF- κβ signalling pathway (FDR = 2.2E−02). Since these two pathways were also the top enriched pathways in the DEG enrichment analysis, we identified them as key pathways. Gene immune function network analysis was performed, and 8 GO terms were determined to be significantly enriched: T cell receptor signalling pathway (FDR = 1.65E−08), neutrophil mediated immunity (FDR =7.37E−05), B cell mediated immunity (FDR = 1.84E−07), regulation of alpha-beta T cell activation (FDR = 3.46E−06), positive regulation of T cell proliferation (FDR = 3.65E−06), regulation of leukocyte differentiation (FDR = 4.12E−06), positive regulation of antigen receptor-mediated signalling pathway (FDR = 5.92E−06), positive regulation of lymphocyte proliferation (FDR = 1.18E−05) (Fig. 6B).

**Figure 7:**
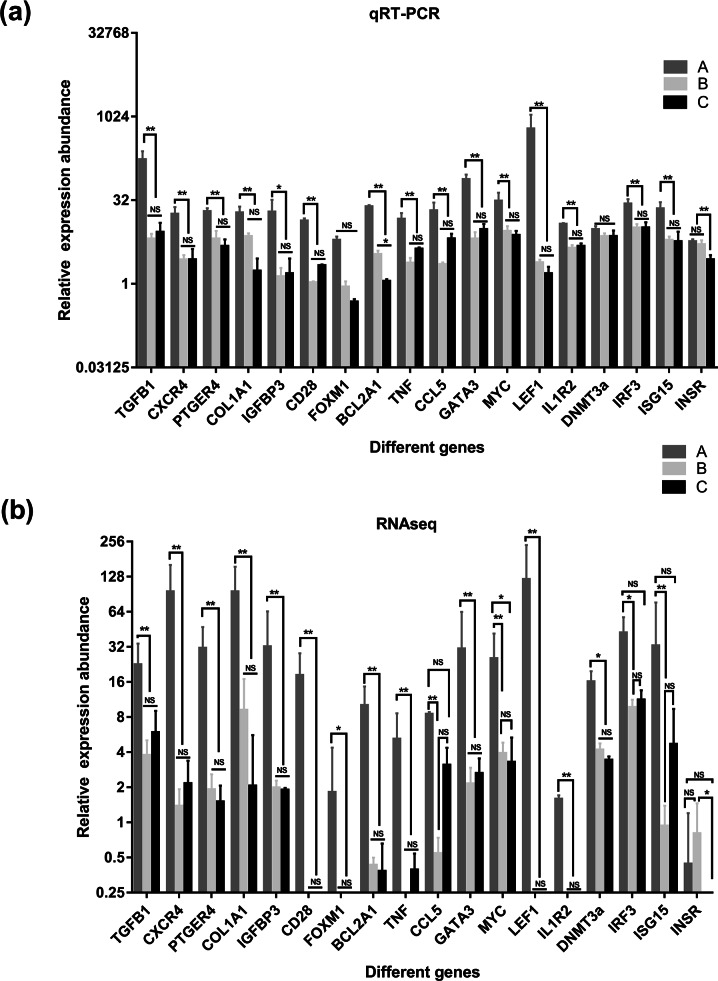
Comparison of relative expression of DEGs between RNA-Seq and real-time PCR results. (A) the results of qRT-PCR, (B) the results of qRT-PCR RNAseq. A were the samples for goat of 1-month-old (group A), B were the samples for goat of 12-month-old (group B), C were the samples for goat of 24-month-old (group C). Significant differences are indicated by * and ** (* indicates *p* < 0.01, ** indicates *p* < 0.05), NS indicates there is no significant differences.

### Validation of DEGs using RT-qPCR

To verify the accuracy of the sequencing and analysis results, 18 differentially expressed mRNAs were selected for RT-qPCR validation (Fig. 7). The RT-qPCR validation results for most selected genes were consistent with the RNA-seq results; only XM_018055547.1 of the DNA (cytosinE-5)-methyltransferase 3A (DNMT3a) gene did not show differential expression in the RT-qPCR analysis. In addition, the forkhead box protein M1 (FOXM1) and interleukin 1 receptor type 2 (IL1R2) genes, which were not expressed in the salivary glands of the goats in group B and group C based on the sequencing data, were detected by RT-qPCR in these two groups.

## Discussion

The growth and development of the submandibular gland is carried out under epithelial-mesenchymal interaction. The process is regulated by a complex cascade consisting of various signaling molecules, including growth/differentiation factors, receptors, cell adhesion molecules, extracellular matrix (ECM) proteins (Wang & Laurie, 2004). In recent years, the function of the submandibular gland gene is gradually discovered. RDH10-mediated metabolism of Vitamin A generates RA that activates RA signaling within the developing SMG, and that the RA signaling directly regulates embryonic growth and morphogenesis of the glan (Wright et al., 2015). Insulin-like growth factor-I (IGF-I), expressed in submandibular gland, may contribute to maintain the cell number, the expression and distribution of tight junction proteins and the paracellular barrier functions of SMIE cells. IGF-I system may participates in the maintenance of viability and the functions of submandibular gland cells (Mitsui et al., 2010). The expression of some of these genes is age-related (Baliu-Piqué et al., 2019). With the change of gene expression, the function of submandibular gland may also be changed. PACE4, a member of subtilisin-like proprotein convertases (SPCs), is critical for the development of submandibular gland. The expression of PACE4 changed during the gestation in rat, and at day 13.5 to 16, the expression level continued to decrease, which may indicate that it’s essential to induce epithelial cell growth during the early stages of bud growth in the submandibular gland (Akamatsu, 2010). The expression of Phospholipase Cβ3 (PLCβ3), which governed the Ca^2+^ signaling, was detected in submandibular gland of mice in newborn and postnatal 2 week by immunoblot, while no expression in postnatal 4 week and 8 week. This indicated the possibility that the differentiation of ductal basal cells into apical cells was weakened (Rawangwong et al., 2020).

Currently, a variety of studies focused on digestion and cell differentiation of submandibular glands. Studies on immune function of submandibular glands have been formed in mice, rat, and human (Carpenter et al., 2004; Salo, Ylikoski & Uusitalo, 1993), however, data on goats are very scarce. Immune-related genes were identified through transcriptome analysis in siamese fighting fish, chicken, dairy cows and giant panda (Amparyup et al., 2020; Guo et al., 2020; Wu et al., 2020; Wang et al., 2020). To understand the pathways and key regulatory genes involved in the immune regulation-related functions of goat submandibular glands at different developmental stages, high-throughput transcriptome sequencing was used in the present study to detect gene expression levels in these glands in goats at three developmental stages. In all of the nine samples, the bases of Q30 or above accouted for more than 94% of the total base of, and Q30 or above accouted for more than 98%. The result of alignment with reference genome showed that over 80% of the identified genes for the nine libraries could be mapped to the goat reference genome, the lowest mapping rate was 83.76%. In previous studies on mammary glands and on goat skin, the mapping haves have been no more than 80%, which indicated that the sequencing quality was high (Xiong et al., 2020; Lin et al., 2015).

We further screened the DEGs in the submandibular glands of the goats at three different stages of growth and development. There were 2,706 and 2,525 DEGs in group A vs. B and A vs. C, respectively. However, there were only 52 DEGs in group B vs. C. Compared with group A, 2,477 and 2,327 genes were downregulated in groups B and C, respectively, which indicated that the gene expression in goat submandibular glands was relatively stable after 12 months of age. This might be related to the complete growth and development of 12- and 24-month-old goats. It has previously been reported that in goats, the thymic mass and cellularity increased after birth and decreased in adults (Baliu-Piqué et al., 2018). Our results also indicated a similar age-dependent manner in the development of submandibular glands.

To investigate the functional distribution of these DEGs, all DEGs were further analysed by GO and KEGG enrichment (Li et al., 2019). The GO results indicated that the most significant enriched term was the immune system process term (Figs. 2 and 3). We observed that 218 DEGs in group A vs. B were annotated into this term, and 214 of them were downregulated. In group A vs. C, 186 DEGs were annotated into this term, and 183 of them were downregulated. DEGs in group B vs. C were not significantly enriched for this term. Almost all of the DEGs were significantly enriched in immune-related terms, including leukocyte activation, lymphocyte activation, T cell activation, regulation of immune system process, regulation of immune response, regulation of leukocyte activation, regulation of lymphocyte activation, and immune response, which were related to immunity and disease resistance (Butler et al., 2009; Mohr & Siegrist, 2016; Morein, Blomqvist & Hu, 2007; Zens, Connors & Farber, 2017; Scheerlinck, 2016). Additionally, the 10 key genes predicted through PPI network analysis were annotated into eight immune-related GO terms (Fig. 5B). The results indicated that the submandibular glands may play vital roles in the innate immune system as the frontier line of immune defence in goat kids and weaken in adolescent and adult goats.

**Figure 8:**
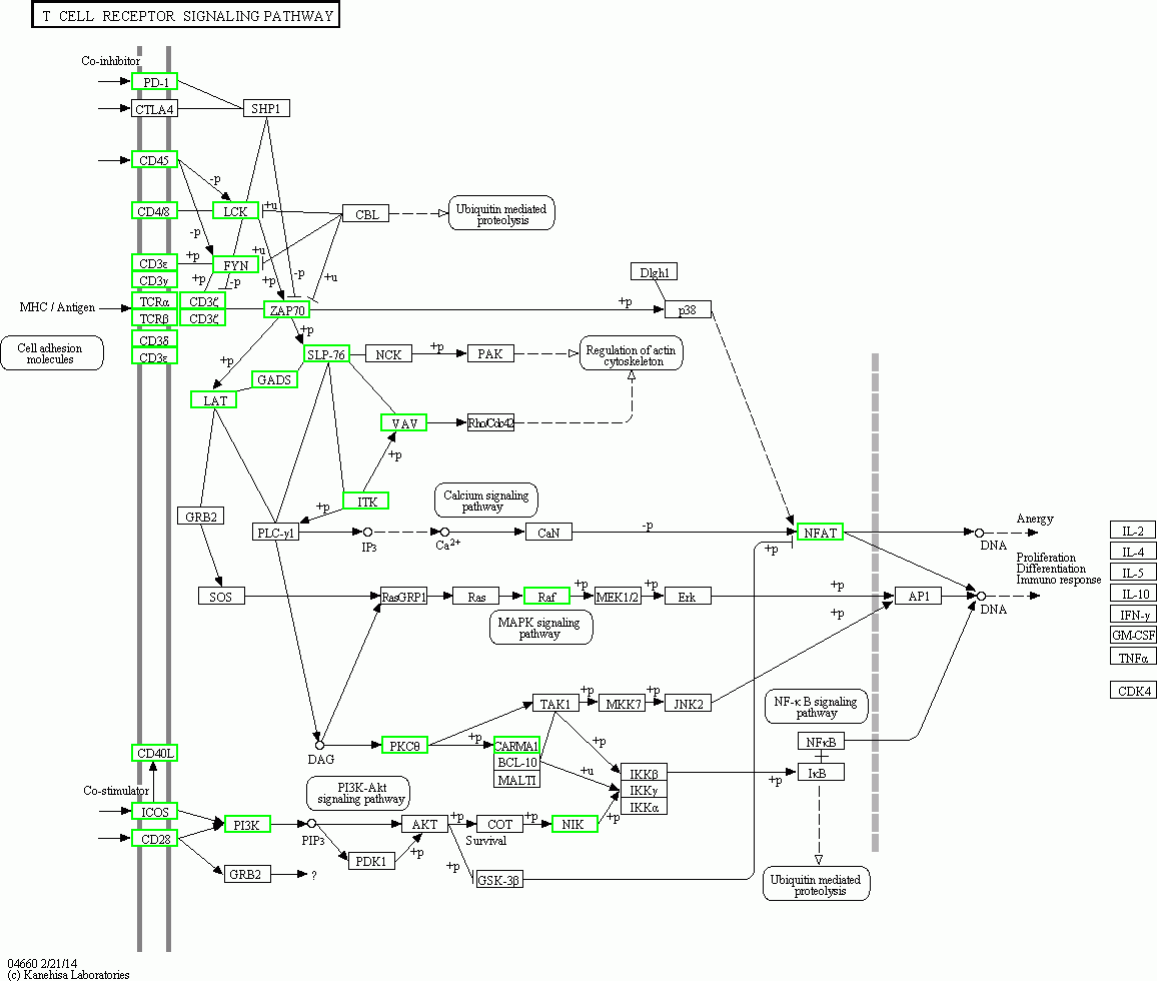
Pathway map of TCR siganalling pathway in KEGG. The green boxes represent down-regulated genes.

The KEGG pathway analysis results showed that the T cell receptor (TCR) signalling pathway and NF-κβ were two significantly enriched pathways. In the TCR signalling pathway, CD45, which is encoded by *PTPRC,* is an evolutionarily highly conserved receptor protein tyrosine phosphatase and is a central player in promoting T cell activation and development (Rheinländer, Schraven & Bommhardt, 2018). CD45 deficiency results in T- and B-lymphocyte dysfunction in the form of severe combined immune deficiency (Courtney, Lo & Weiss, 2018; Chakraborty & Weiss, 2014). Lck is a member of the Src family of nonreceptor protein tyrosine kinases that phosphorylate ZAP70 and ITK (Rohrs, Wang & Finley, 2016; Singh, Kashyap & Silakari, 2018). Zeta-chain associated protein kinase (ZAP70), a member of the Syk family kinase, is predominantly a tyrosine kinase protein that is involved in T cell receptor (TCR) signalling initiation and subsequent T cell activation. TCR signalling is orchestrated initially by coordinated interactions of the Lck and ZAP70 protein tyrosine kinases (Chanet al., 1992; Balagopalan et al., 2010; Zhang et al., 2000; Ahmed et al., 2005; Amin, Parker & Mann, 2008). Our results showed that in adolescent and adult goats, the T cell receptor signalling pathway may suggest weakened submandibular glands. In this study, the expression of *CD45, LCK, ITK* and *ZAP70,* which were also predicted to be potential key regulatory genes by PPI network analysis, was significantly downregulated in the TCR signalling pathway (Fig. 8) in adolescent and adult goats (Courtney et al. 2017). This may suggest the reason why the immune function of submandibular glands weakened with growth. At the same time, the expression of CD28 was downregulated in adolescent and adult goats in our results. The CD28 receptor provides a critical second signal alongside TCR ligation for naive T cell activation (Esensten et al., 2016). Thus, CD28 may also weaken T cell activation by the role of the TCR signalling pathway.

Three key regulatory genes (LCK, ZAP70 and SYK) were significantly enriched in another potential key regulatory pathway, the NF-kappa B signalling pathway. The canonical N-κβ signalling pathway is regarded as the central regulator of the inflammatory response in normal physiology (Cildir, Low & Tergaonkar, 2016). Phosphorylation of LCK and ZAP70 activates the LCK-NF-κB axis (Huang et al., 2011). SYK can activate the NF-κB pathway, which subsequently triggers excessive production of chemokines and cytokines, leading to inflammatory responses (Lee, Chain & Cho, 2009). Similar to the TCR signalling pathway, in the NF-κβ signalling pathway, all three key regulatory genes were downregulated in adolescent and adult goats. Taken together, our findings indicate that the downregulated DEGs related to the TCR signalling pathway and NF-κβ signalling pathway activation may be responsible for immune function in the submandibular gland in goat kids. Therefore, the function of these genes deserves further attention. The expression levels of IgA, IgE, IgD, IgM and IgG in the submandibular glands of adolescent and adult goats were significantly upregulated (Table 3). Based on the analysis results, we hypothesize that the submandibular glands may be one of the important immunological organs several months after birth and that immunoglobulins play a major immunological role later in life.

Additionally, we found that 8 antibacterial peptide (AMP) genes (BAC7.5, MAP28, BAC5, MAP34-B, CATHL1A, LOC102171106, LOC102169231 and LOC102170003) enriched in the salivary secretion pathway were downregulated in groups B and C compared with group A. Animal AMPs, which form a class of peptides of animal origin that show broad-spectrum antimicrobial activity (Mwangi et al., 2019), are essential components of the innate immune system (Young-Speirs et al., 2018; Mwangi et al., 2019; Panteleev et al., 2018). In goats, several AMPs have been identified and characterized from bone marrow, neutrophils, epithelia and mammary glands (Young-Speirs et al., 2018; Bagella, Scocchi & Zanetti, 1995; Kościuczuk et al., 2012; Zaiou & Gallo, 2002), and no AMP data are available in submandibular glands to date. The 8 AMPs that have high antimicrobial activity against gram-negative bacteria were also enriched in the tuberculosis pathway. DEG analysis revealed that the expression levels of the above 8 AMPs in the submandibular glands of goat kids were higher than those in adolescent and adult goats, indicating that the 8 AMPs may play an important role in the immune system in goat kids to fully understand the potential functions of cathelicidins in goats, we need to precisely understand their in vivo roles.

In conclution, GO and KEGG analyses explained the immune function of the submandibular gland in goat kids, Molecular-assisted breeding techniques have been used for goat breeding (Dodds, McEwan & Davis, 2007). The 10 potential key regulatory genes found in our research might contribute to the enhancement of molecular breeding for the improvement of goat immunity.

## Conclusions

High-throughput transcriptome sequencing was conducted on nine submandibular gland samples from goats at 1, 12 and 24 months of age (three replicates per age). DEG analysis revealed the existence of 2,706, 2,525 and 52 differentially expressed mRNAs between 1-month-old and 12-month-old goats, between 1-month-old and 24-month-old goats, and between 12-month-old and 24-month-old goats, respectively. GO and KEGG enrichment analyses of the DEGs revealed that the immune system GO term, TCR signalling pathway and the NF-κβ signalling pathway were significantly enriched. Ten key regulator genes identified by the PPI network were identified as potential key regulatory genes. In this research, we revealed the immune functions of goat submandibular glands at different ages and analysed the key genes and signalling pathways. To further understand the immune mechanism of submandibular glands, we need to further study the exact function of key regulator genes at the molecular level.

##  Supplemental Information

10.7717/peerj.9947/supp-1 Table S1 Reads of The Nine Libraries A1-L, A2-L, A3-L were the samples for goats of 1-month-old (group A), B3-L, B4-L, B5-L were the samples for goats of 12-month-old (group B ), C2-L, C3-L, C5-L were the samples for goats of 24-month-old (group C).

10.7717/peerj.9947/supp-2 Table S2 Statistics of known mRNAs and new transcripts of different age groups A1-L, A2-L, A3-L were the samples for goats of 1-month-old (group A), B3-L, B4-L, B5-L were the samples for goats of 12-month-old (group B ), C2-L, C3-L, C5-L were the samples for goats of 24-month-old (group C).

10.7717/peerj.9947/supp-3 Table S3 Differential Expression mRNA A-vs-B A1-L, A2-L, A3-L were the samples for goats of 1-month-old (group A), B3-L, B4-L, B5-L were the samples for goats of 12-month-old (group B )

10.7717/peerj.9947/supp-4 Table S4 Differential Expression mRNA A-vs-C A1-L, A2-L, A3-L were the samples for goats of 1-month-old (group A), C2-L, C3-L, C5-L were the samples for goats of 24-month-old (group C).

10.7717/peerj.9947/supp-5 Table S5 Differential Expression mRNA B-vs-C 3-L, B4-L, B5-L were the samples for goats of 12-month-old (group B ), C2-L, C3-L, C5-L were the samples for goats of 24-month-old (group C).

10.7717/peerj.9947/supp-6 Table S6 Statistics of known and new transcripts in each sample A1-L, A2-L, A3-L were the samples for goats of 1-month-old (group A), B3-L, B4-L, B5-L were the samples for goats of 12-month-old (group B ), C2-L, C3-L, C5-L were the samples for goats of 24-month-old (group C).

10.7717/peerj.9947/supp-7 Table S7 Significantly enriched GO terms of DEGs between group A and B

10.7717/peerj.9947/supp-8 Table S8 Significantly enriched GO terms of DEGs between group A and C

## Additional Information and Declarations

### Data Availability

The following information was supplied regarding data availability: All data is available at the Gene Expression Omnibus (GEO): PRJNA603495 and GSE144368. The dataset and the concluding report of goat submandibular glands are also available as Supplemental Files.
