# Supplementary material for: Transcriptome analysis reveals potential immune function-related regulatory genes/pathways of female Lubo goat submandibular glands at different developmental stages
Source: PeerJ. 2020 Oct 7;8:e9947. doi: 10.7717/peerj.9947 (PMC7547598; doi:10.7717/peerj.9947)
Supplement: Table S1 — A1-L, A2-L, A3-L were the samples for goats of 1-month-old (group A), B3-L, B4-L, B5-L were the samples for goats of 12-month-old (group B ), C2-L, C3-L, C5-L were the samples for goats of 24-month-old (group C). [file peerj-08-9947-s001.docx]

**[Table S1:](file:///C:\\Users\\Administrator\\Desktop\\%E5%85%A8%E8%BD%AC%E5%BD%95%E7%BB%84%E7%BB%93%E9%A2%98%E6%8A%A5%E5%91%8A%20%E5%AE%8C%E6%95%B4%E7%89%88%2020190305\\GDR4449-Capra_hircus-9-lncRNA-result\\GDR4449-Capra_hircus_-9-lncRNA-result\\src\\doc\\data_stat.html#Reads%20过滤信息统计表说明)**

**[Reads of The Nine Libraries](file:///C:\\Users\\Administrator\\Desktop\\%E5%85%A8%E8%BD%AC%E5%BD%95%E7%BB%84%E7%BB%93%E9%A2%98%E6%8A%A5%E5%91%8A%20%E5%AE%8C%E6%95%B4%E7%89%88%2020190305\\GDR4449-Capra_hircus-9-lncRNA-result\\GDR4449-Capra_hircus_-9-lncRNA-result\\src\\doc\\data_stat.html#Reads%20过滤信息统计表说明)**

| Sample | Clean Reads Num | HQ Clean Reads | Num  (%) | Adapter  (%) | Low Quality  (%) | Poly A  (%) | N  (%) |
| --- | --- | --- | --- | --- | --- | --- | --- |
| A1-L | 96998078 | 96072674 | 99.05% | 343758 (0.35%) | 581052  (0.6%) | 296 (0%) | 1 (0%) |
| A2-L | 88331132 | 87245234 | 98.77% | 349930  (0.4%) | 735546 (0.83%) | 211 (0%) | 0 (0%) |
| A3-L | 114954540 | 113739046 | 98.94% | 419996 (0.37%) | 794754 (0.69%) | 371 (0%) | 1 (0%) |
| B3-L | 104960244 | 103951102 | 99.04% | 329876 (0.31%) | 678704 (0.65%) | 281 (0%) | 0 (0%) |
| B4-L | 86933502 | 86181538 | 99.14% | 250188 (0.29%) | 501370 (0.58%) | 203 (0%) | 0 (0%) |
| B5-L | 97842854 | 96775574 | 98.91% | 377264 (0.39%) | 689494 (0.7%) | 260 (0%) | 1 (0%) |
| C2-L | 88505540 | 87599270 | 98.98% | 259406 (0.29%) | 646438 (0.73%) | 213 (0%) | 0 (0%) |
| C3-L | 93855424 | 92790270 | 98.87% | 310694 (0.33%) | 753986 (0.8%) | 235 (0%) | 2 (0%) |
| C5-L | 90614558 | 89431754 | 98.69% | 353238 (0.39%) | 829104 (0.91%) | 231 (0%) | 0 (0%) |
| summary | 862995872 | 853786462 | / | / | / | / | / |
