# Supplementary material for: Transcriptome analysis reveals potential immune function-related regulatory genes/pathways of female Lubo goat submandibular glands at different developmental stages
Source: PeerJ. 2020 Oct 7;8:e9947. doi: 10.7717/peerj.9947 (PMC7547598; doi:10.7717/peerj.9947)
Supplement: Table S2 — A1-L, A2-L, A3-L were the samples for goats of 1-month-old (group A), B3-L, B4-L, B5-L were the samples for goats of 12-month-old (group B ), C2-L, C3-L, C5-L were the samples for goats of 24-month-old (group C). [file peerj-08-9947-s002.docx]

**Table S2:**

**Statistics of known and new transcripts in each group**

| Sample Name | Known mRNA Num | New mRNA Num | All mRNA Num |
| --- | --- | --- | --- |
| A1-L | 27763 (65.04%) | 7355 | 35118 |
| A2-L | 26710 (62.57%) | 6778 | 33488 |
| A3-L | 28581 (66.96%) | 7549 | 36130 |
| B3-L | 26068 (61.07%) | 6821 | 32889 |
| B4-L | 25347 (59.38%) | 6544 | 31891 |
| B5-L | 25819 (60.49%) | 6658 | 32477 |
| C2-L | 25075 (58.74%) | 6504 | 31579 |
| C3-L | 23988 (56.20%) | 6107 | 30095 |
| C5-L | 23946 (56.10%) | 6080 | 30026 |
