# Supplementary material for: Transcriptome analysis reveals potential immune function-related regulatory genes/pathways of female Lubo goat submandibular glands at different developmental stages
Source: PeerJ. 2020 Oct 7;8:e9947. doi: 10.7717/peerj.9947 (PMC7547598; doi:10.7717/peerj.9947)
Supplement: Table S6 — A1-L, A2-L, A3-L were the samples for goats of 1-month-old (group A), B3-L, B4-L, B5-L were the samples for goats of 12-month-old (group B ), C2-L, C3-L, C5-L were the samples for goats of 24-month-old (group C). [file peerj-08-9947-s006.docx]

**Table S7:**

**Statistics of known and new transcripts in each sample**

| **Sample Name** | **Known mRNA Num** | **New mRNA Num** | **All mRNA Num** |
| --- | --- | --- | --- |
| **A1-L** | 27763 (65.04%) | 7355 | 35118 |
| **A2-L** | 26710 (62.57%) | 6778 | 33488 |
| **A3-L** | 28581 (66.96%) | 7549 | 36130 |
| **B3-L** | 26068 (61.07%) | 6821 | 32889 |
| **B4-L** | 25347 (59.38%) | 6544 | 31891 |
| **B5-L** | 25819 (60.49%) | 6658 | 32477 |
| **C2-L** | 25075 (58.74%) | 6504 | 31579 |
| **C3-L** | 23988 (56.20%) | 6107 | 30095 |
| **C5-L** | 23946 (56.10%) | 6080 | 30026 |

A1-L、 A2-L、 A3-L were the samples for goat of 1-month-old (group A), B3-L、B4-L、B5-L were the samples for goat of 12-month-old (group B ), C2-L、C3-L、C5-L were the samples for goat of 24-month-old (group C).
