# Supplementary material for: Transcriptome analysis reveals potential immune function-related regulatory genes/pathways of female Lubo goat submandibular glands at different developmental stages
Source: PeerJ. 2020 Oct 7;8:e9947. doi: 10.7717/peerj.9947 (PMC7547598; doi:10.7717/peerj.9947)
Supplement: Table S7 [file peerj-08-9947-s007.docx]

**Table S8:**

**Significantly enriched GO terms of DEGs between group A and B**

| **Type** | **Term** | **FDR** |
| --- | --- | --- |
| Biological Process | GO:0002376 immune system process | 6.26E-38 |
| Biological Process | GO:0045321 leukocyte activation | 5.44E-33 |
| Biological Process | GO:0046649 lymphocyte activation | 9.28E-32 |
| Biological Process | GO:0001775 cell activation | 2.57E-31 |
| Biological Process | GO:0070486 leukocyte aggregation | 1.04E-24 |
| Biological Process | GO:0034109 homotypic cell-cell adhesion | 1.24E-24 |
| Biological Process | GO:0007159 leukocyte cell-cell adhesion | 3.00E-24 |
| Biological Process | GO:0042110 T cell activation | 2.99E-22 |
| Biological Process | GO:0070489 T cell aggregation | 2.99E-22 |
| Biological Process | GO:0071593 lymphocyte aggregation | 2.99E-22 |
| Biological Process | GO:0016337 single organismal cell-cell adhesion | 2.51E-21 |
| Biological Process | GO:0002682 regulation of immune system process | 4.79E-21 |
| Biological Process | GO:0030098 lymphocyte differentiation | 1.10E-13 |
| Biological Process | GO:0050776 regulation of immune response | 1.10E-13 |
| Biological Process | GO:0050865 regulation of cell activation | 4.11E-13 |
| Biological Process | GO:0098602 single organism cell adhesion | 4.18E-13 |
| Biological Process | GO:0098609 cell-cell adhesion | 8.55E-13 |
| Biological Process | GO:0002684 positive regulation of immune system process | 2.12E-12 |
| Biological Process | GO:0009605 response to external stimulus | 2.23E-12 |
| Biological Process | GO:0002694 regulation of leukocyte activation | 2.27E-12 |
| Biological Process | GO:0051276 chromosome organization | 2.27E-12 |
| Biological Process | GO:0007049 cell cycle | 3.64E-12 |
| Biological Process | GO:0007155 cell adhesion | 3.92E-12 |
| Biological Process | GO:0051249 regulation of lymphocyte activation | 7.64E-12 |
| Biological Process | GO:0002252 immune effector process | 3.55E-11 |
| Biological Process | GO:0045619 regulation of lymphocyte differentiation | 3.72E-11 |
| Biological Process | GO:0022610 biological adhesion | 3.85E-11 |
| Biological Process | GO:0030217 T cell differentiation | 5.01E-11 |
| Biological Process | GO:0043207 response to external biotic stimulus | 6.84E-11 |
| Biological Process | GO:0002521 leukocyte differentiation | 6.86E-11 |
| Biological Process | GO:0009607 response to biotic stimulus | 8.81E-11 |
| Biological Process | GO:0002520 immune system development | 1.24E-10 |
| Biological Process | GO:0022402 cell cycle process | 1.86E-10 |
| Biological Process | GO:0034110 regulation of homotypic cell-cell adhesion | 2.54E-10 |
| Biological Process | GO:0051707 response to other organism | 2.59E-10 |
| Biological Process | GO:0048534 hematopoietic or lymphoid organ development | 1.24E-09 |
| Biological Process | GO:0006996 organelle organization | 1.98E-09 |
| Biological Process | GO:0050778 positive regulation of immune response | 2.13E-09 |
| Biological Process | GO:0034728 nucleosome organization | 2.23E-09 |
| Biological Process | GO:0016043 cellular component organization | 3.55E-09 |
| Biological Process | GO:0071824 protein-DNA complex subunit organization | 9.35E-09 |
| Biological Process | GO:0071840 cellular component organization or biogenesis | 1.16E-08 |
| Biological Process | GO:0006325 chromatin organization | 1.27E-08 |
| Biological Process | GO:0030097 hemopoiesis | 2.23E-08 |
| Biological Process | GO:1902105 regulation of leukocyte differentiation | 2.41E-08 |
| Biological Process | GO:0006950 response to stress | 2.44E-08 |
| Biological Process | GO:0022407 regulation of cell-cell adhesion | 3.41E-08 |
| Biological Process | GO:0043933 macromolecular complex subunit organization | 4.08E-08 |
| Biological Process | GO:0042113 B cell activation | 5.95E-08 |
| Biological Process | GO:0006955 immune response | 6.36E-08 |
| Biological Process | GO:0046631 alpha-beta T cell activation | 8.54E-08 |
| Biological Process | GO:0070661 leukocyte proliferation | 8.89E-08 |
| Biological Process | GO:0051240 positive regulation of multicellular organismal process | 1.08E-07 |
| Biological Process | GO:0009617 response to bacterium | 1.12E-07 |
| Biological Process | GO:0050854 regulation of antigen receptor-mediated signaling pathway | 1.24E-07 |
| Biological Process | GO:1903706 regulation of hemopoiesis | 1.34E-07 |
| Biological Process | GO:0050863 regulation of T cell activation | 1.49E-07 |
| Biological Process | GO:1903037 regulation of leukocyte cell-cell adhesion | 1.49E-07 |
| Biological Process | GO:0050851 antigen receptor-mediated signaling pathway | 1.75E-07 |
| Biological Process | GO:0032943 mononuclear cell proliferation | 3.44E-07 |
| Biological Process | GO:0046651 lymphocyte proliferation | 5.38E-07 |
| Biological Process | GO:0001819 positive regulation of cytokine production | 6.08E-07 |
| Biological Process | GO:0034112 positive regulation of homotypic cell-cell adhesion | 8.71E-07 |
| Biological Process | GO:0050896 response to stimulus | 9.52E-07 |
| Biological Process | GO:0071822 protein complex subunit organization | 1.01E-06 |
| Biological Process | GO:0048583 regulation of response to stimulus | 1.44E-06 |
| Biological Process | GO:0002429 immune response-activating cell surface receptor signaling pathway | 1.59E-06 |
| Biological Process | GO:0002443 leukocyte mediated immunity | 1.62E-06 |
| Biological Process | GO:0051262 protein tetramerization | 1.76E-06 |
| Biological Process | GO:0045580 regulation of T cell differentiation | 2.18E-06 |
| Biological Process | GO:0030155 regulation of cell adhesion | 2.28E-06 |
| Biological Process | GO:0050900 leukocyte migration | 2.63E-06 |
| Biological Process | GO:0051239 regulation of multicellular organismal process | 2.81E-06 |
| Biological Process | GO:0000278 mitotic cell cycle | 2.85E-06 |
| Biological Process | GO:0040011 locomotion | 2.98E-06 |
| Biological Process | GO:0036037 CD8-positive, alpha-beta T cell activation | 3.89E-06 |
| Biological Process | GO:1903047 mitotic cell cycle process | 4.12E-06 |
| Biological Process | GO:0002696 positive regulation of leukocyte activation | 4.31E-06 |
| Biological Process | GO:0002253 activation of immune response | 4.47E-06 |
| Biological Process | GO:0050670 regulation of lymphocyte proliferation | 5.92E-06 |
| Biological Process | GO:0022409 positive regulation of cell-cell adhesion | 6.28E-06 |
| Biological Process | GO:0048584 positive regulation of response to stimulus | 6.64E-06 |
| Biological Process | GO:0050867 positive regulation of cell activation | 6.71E-06 |
| Biological Process | GO:0045058 T cell selection | 7.01E-06 |
| Biological Process | GO:0070229 negative regulation of lymphocyte apoptotic process | 7.61E-06 |
| Biological Process | GO:2000107 negative regulation of leukocyte apoptotic process | 7.61E-06 |
| Biological Process | GO:0006935 chemotaxis | 7.64E-06 |
| Biological Process | GO:0042098 T cell proliferation | 7.64E-06 |
| Biological Process | GO:0042330 taxis | 7.64E-06 |
| Biological Process | GO:0050794 regulation of cellular process | 8.78E-06 |
| Biological Process | GO:0001817 regulation of cytokine production | 9.95E-06 |
| Biological Process | GO:0031347 regulation of defense response | 1.04E-05 |
| Biological Process | GO:0050789 regulation of biological process | 1.24E-05 |
| Biological Process | GO:0006952 defense response | 1.27E-05 |
| Biological Process | GO:0032944 regulation of mononuclear cell proliferation | 1.63E-05 |
| Biological Process | GO:0001906 cell killing | 1.86E-05 |
| Biological Process | GO:0006259 DNA metabolic process | 1.91E-05 |
| Biological Process | GO:2000106 regulation of leukocyte apoptotic process | 1.92E-05 |
| Biological Process | GO:0000280 nuclear division | 2.40E-05 |
| Biological Process | GO:0002697 regulation of immune effector process | 2.46E-05 |
| Biological Process | GO:0035710 CD4-positive, alpha-beta T cell activation | 2.46E-05 |
| Biological Process | GO:0002768 immune response-regulating cell surface receptor signaling pathway | 2.93E-05 |
| Biological Process | GO:0002819 regulation of adaptive immune response | 3.38E-05 |
| Biological Process | GO:0045785 positive regulation of cell adhesion | 3.55E-05 |
| Biological Process | GO:0070663 regulation of leukocyte proliferation | 3.90E-05 |
| Biological Process | GO:0046637 regulation of alpha-beta T cell differentiation | 4.34E-05 |
| Biological Process | GO:0009620 response to fungus | 4.71E-05 |
| Biological Process | GO:0032743 positive regulation of interleukin-2 production | 4.71E-05 |
| Biological Process | GO:0002757 immune response-activating signal transduction | 4.87E-05 |
| Biological Process | GO:0051251 positive regulation of lymphocyte activation | 5.06E-05 |
| Biological Process | GO:0048285 organelle fission | 5.29E-05 |
| Biological Process | GO:0031577 spindle checkpoint | 5.93E-05 |
| Biological Process | GO:2000026 regulation of multicellular organismal development | 7.62E-05 |
| Biological Process | GO:0048870 cell motility | 8.23E-05 |
| Biological Process | GO:0051674 localization of cell | 8.23E-05 |
| Biological Process | GO:0007051 spindle organization | 8.83E-05 |
| Biological Process | GO:0030595 leukocyte chemotaxis | 9.37E-05 |
| Biological Process | GO:0002822 regulation of adaptive immune response based on somatic recombination of immune receptors built from immunoglobulin superfamily domains | 0.000111649 |
| Biological Process | GO:0032663 regulation of interleukin-2 production | 0.000118478 |
| Biological Process | GO:0048518 positive regulation of biological process | 0.000130383 |
| Biological Process | GO:0006928 movement of cell or subcellular component | 0.000137618 |
| Biological Process | GO:0071621 granulocyte chemotaxis | 0.000158985 |
| Biological Process | GO:0042129 regulation of T cell proliferation | 0.000177509 |
| Biological Process | GO:0046634 regulation of alpha-beta T cell activation | 0.000184802 |
| Biological Process | GO:0002706 regulation of lymphocyte mediated immunity | 0.000187659 |
| Biological Process | GO:0002764 immune response-regulating signaling pathway | 0.000210944 |
| Biological Process | GO:0044763 single-organism cellular process | 0.000227061 |
| Biological Process | GO:0071103 DNA conformation change | 0.000234478 |
| Biological Process | GO:0002821 positive regulation of adaptive immune response | 0.000236307 |
| Biological Process | GO:0002824 positive regulation of adaptive immune response based on somatic recombination of immune receptors built from immunoglobulin superfamily domains | 0.000236307 |
| Biological Process | GO:0097530 granulocyte migration | 0.000236307 |
| Biological Process | GO:0060326 cell chemotaxis | 0.000238722 |
| Biological Process | GO:0002562 somatic diversification of immune receptors via germline recombination within a single locus | 0.00024166 |
| Biological Process | GO:0016444 somatic cell DNA recombination | 0.00024166 |
| Biological Process | GO:0003230 cardiac atrium development | 0.000267729 |
| Biological Process | GO:0065007 biological regulation | 0.000275368 |
| Biological Process | GO:0070228 regulation of lymphocyte apoptotic process | 0.00030778 |
| Biological Process | GO:0002703 regulation of leukocyte mediated immunity | 0.000385493 |
| Biological Process | GO:1902589 single-organism organelle organization | 0.000418059 |
| Biological Process | GO:0097529 myeloid leukocyte migration | 0.000425683 |
| Biological Process | GO:0051726 regulation of cell cycle | 0.000438919 |
| Biological Process | GO:0051704 multi-organism process | 0.000456727 |
| Biological Process | GO:0002705 positive regulation of leukocyte mediated immunity | 0.000498321 |
| Biological Process | GO:0034502 protein localization to chromosome | 0.000498321 |
| Biological Process | GO:0007010 cytoskeleton organization | 0.00052753 |
| Biological Process | GO:0000075 cell cycle checkpoint | 0.000532954 |
| Biological Process | GO:0006897 endocytosis | 0.00054256 |
| Biological Process | GO:0065009 regulation of molecular function | 0.00054256 |
| Biological Process | GO:0002263 cell activation involved in immune response | 0.000562484 |
| Biological Process | GO:0002366 leukocyte activation involved in immune response | 0.000562484 |
| Biological Process | GO:0002708 positive regulation of lymphocyte mediated immunity | 0.000562484 |
| Biological Process | GO:0030154 cell differentiation | 0.000589003 |
| Biological Process | GO:0002274 myeloid leukocyte activation | 0.000598461 |
| Biological Process | GO:0050870 positive regulation of T cell activation | 0.00067615 |
| Biological Process | GO:1903039 positive regulation of leukocyte cell-cell adhesion | 0.00067615 |
| Biological Process | GO:0050864 regulation of B cell activation | 0.00070119 |
| Biological Process | GO:0032508 DNA duplex unwinding | 0.000752246 |
| Biological Process | GO:0000226 microtubule cytoskeleton organization | 0.000864716 |
| Biological Process | GO:0006310 DNA recombination | 0.000907375 |
| Biological Process | GO:0098542 defense response to other organism | 0.000907375 |
| Biological Process | GO:1903650 negative regulation of cytoplasmic transport | 0.000971863 |
| Biological Process | GO:0016477 cell migration | 0.000980157 |
| Biological Process | GO:0043383 negative T cell selection | 0.000982027 |
| Biological Process | GO:0034097 response to cytokine | 0.001135107 |
| Biological Process | GO:0048519 negative regulation of biological process | 0.001135474 |
| Biological Process | GO:0043370 regulation of CD4-positive, alpha-beta T cell differentiation | 0.001150534 |
| Biological Process | GO:2000514 regulation of CD4-positive, alpha-beta T cell activation | 0.001150534 |
| Biological Process | GO:0002449 lymphocyte mediated immunity | 0.001195161 |
| Biological Process | GO:0042127 regulation of cell proliferation | 0.001220731 |
| Biological Process | GO:0002699 positive regulation of immune effector process | 0.001291861 |
| Biological Process | GO:0002200 somatic diversification of immune receptors | 0.001336299 |
| Biological Process | GO:0032387 negative regulation of intracellular transport | 0.001484454 |
| Biological Process | GO:0048513 animal organ development | 0.001707055 |
| Biological Process | GO:0042742 defense response to bacterium | 0.001726106 |
| Biological Process | GO:0043087 regulation of GTPase activity | 0.001795261 |
| Biological Process | GO:0002700 regulation of production of molecular mediator of immune response | 0.001834702 |
| Biological Process | GO:0046823 negative regulation of nucleocytoplasmic transport | 0.001922555 |
| Biological Process | GO:0051983 regulation of chromosome segregation | 0.001922555 |
| Biological Process | GO:0002712 regulation of B cell mediated immunity | 0.002249224 |
| Biological Process | GO:0016568 chromatin modification | 0.002362809 |
| Biological Process | GO:0001909 leukocyte mediated cytotoxicity | 0.002571193 |
| Biological Process | GO:0006338 chromatin remodeling | 0.002687834 |
| Biological Process | GO:0050856 regulation of T cell receptor signaling pathway | 0.002716964 |
| Biological Process | GO:0002714 positive regulation of B cell mediated immunity | 0.003105908 |
| Biological Process | GO:0042743 hydrogen peroxide metabolic process | 0.003105908 |
| Biological Process | GO:0000212 meiotic spindle organization | 0.003518278 |
| Biological Process | GO:0030852 regulation of granulocyte differentiation | 0.003518278 |
| Biological Process | GO:0032875 regulation of DNA endoreduplication | 0.003518278 |
| Biological Process | GO:0006323 DNA packaging | 0.003885042 |
| Biological Process | GO:0080134 regulation of response to stress | 0.003885042 |
| Biological Process | GO:0010564 regulation of cell cycle process | 0.003936514 |
| Biological Process | GO:0010033 response to organic substance | 0.004130013 |
| Biological Process | GO:0031348 negative regulation of defense response | 0.004239981 |
| Biological Process | GO:0003209 cardiac atrium morphogenesis | 0.004277149 |
| Biological Process | GO:0050855 regulation of B cell receptor signaling pathway | 0.004277149 |
| Biological Process | GO:0009611 response to wounding | 0.004368347 |
| Biological Process | GO:0065008 regulation of biological quality | 0.004433885 |
| Biological Process | GO:0050793 regulation of developmental process | 0.005081573 |
| Biological Process | GO:0040017 positive regulation of locomotion | 0.005148542 |
| Biological Process | GO:0030888 regulation of B cell proliferation | 0.00522755 |
| Biological Process | GO:0002285 lymphocyte activation involved in immune response | 0.005428941 |
| Biological Process | GO:0002831 regulation of response to biotic stimulus | 0.005428941 |
| Biological Process | GO:0048520 positive regulation of behavior | 0.005428941 |
| Biological Process | GO:0050921 positive regulation of chemotaxis | 0.005428941 |
| Biological Process | GO:0001818 negative regulation of cytokine production | 0.005498201 |
| Biological Process | GO:0035712 T-helper 2 cell activation | 0.005908953 |
| Biological Process | GO:0071173 spindle assembly checkpoint | 0.006080677 |
| Biological Process | GO:0006305 DNA alkylation | 0.006113644 |
| Biological Process | GO:0002250 adaptive immune response | 0.006336274 |
| Biological Process | GO:0002460 adaptive immune response based on somatic recombination of immune receptors built from immunoglobulin superfamily domains | 0.006336274 |
| Biological Process | GO:0071624 positive regulation of granulocyte chemotaxis | 0.006435079 |
| Biological Process | GO:0050790 regulation of catalytic activity | 0.00645899 |
| Biological Process | GO:0008277 regulation of G-protein coupled receptor protein signaling pathway | 0.006802068 |
| Biological Process | GO:0030183 B cell differentiation | 0.006816749 |
| Biological Process | GO:0045191 regulation of isotype switching | 0.006816749 |
| Biological Process | GO:0050663 cytokine secretion | 0.006816749 |
| Biological Process | GO:0050848 regulation of calcium-mediated signaling | 0.007326194 |
| Biological Process | GO:0051128 regulation of cellular component organization | 0.007537419 |
| Biological Process | GO:0048869 cellular developmental process | 0.007912346 |
| Biological Process | GO:0007059 chromosome segregation | 0.008070315 |
| Biological Process | GO:1903649 regulation of cytoplasmic transport | 0.008285776 |
| Biological Process | GO:0032101 regulation of response to external stimulus | 0.008343582 |
| Biological Process | GO:0051052 regulation of DNA metabolic process | 0.00852502 |
| Biological Process | GO:0044319 wound healing, spreading of cells | 0.009272188 |
| Biological Process | GO:0090504 epiboly | 0.009272188 |
| Biological Process | GO:0090505 epiboly involved in wound healing | 0.009272188 |
| Biological Process | GO:0048731 system development | 0.009825998 |
| Biological Process | GO:0002685 regulation of leukocyte migration | 0.010017291 |
| Biological Process | GO:0033077 T cell differentiation in thymus | 0.010027645 |
| Biological Process | GO:0060413 atrial septum morphogenesis | 0.010027645 |
| Biological Process | GO:0071594 thymocyte aggregation | 0.010027645 |
| Biological Process | GO:0031647 regulation of protein stability | 0.010242515 |
| Biological Process | GO:0051051 negative regulation of transport | 0.010242515 |
| Biological Process | GO:0044770 cell cycle phase transition | 0.010363705 |
| Biological Process | GO:0044772 mitotic cell cycle phase transition | 0.010363705 |
| Biological Process | GO:0010993 regulation of ubiquitin homeostasis | 0.01069722 |
| Biological Process | GO:0050878 regulation of body fluid levels | 0.010982733 |
| Biological Process | GO:0045595 regulation of cell differentiation | 0.010989712 |
| Biological Process | GO:0010543 regulation of platelet activation | 0.011498732 |
| Biological Process | GO:0002637 regulation of immunoglobulin production | 0.01237058 |
| Biological Process | GO:0032434 regulation of proteasomal ubiquitin-dependent protein catabolic process | 0.012534801 |
| Biological Process | GO:0043900 regulation of multi-organism process | 0.012534801 |
| Biological Process | GO:0044707 single-multicellular organism process | 0.012534801 |
| Biological Process | GO:0045830 positive regulation of isotype switching | 0.012534801 |
| Biological Process | GO:0045911 positive regulation of DNA recombination | 0.012534801 |
| Biological Process | GO:0050871 positive regulation of B cell activation | 0.012534801 |
| Biological Process | GO:0061640 cytoskeleton-dependent cytokinesis | 0.012534801 |
| Biological Process | GO:0010604 positive regulation of macromolecule metabolic process | 0.012626357 |
| Biological Process | GO:0002440 production of molecular mediator of immune response | 0.013763 |
| Biological Process | GO:0007599 hemostasis | 0.014174417 |
| Biological Process | GO:0033043 regulation of organelle organization | 0.014232704 |
| Biological Process | GO:0006954 inflammatory response | 0.014246238 |
| Biological Process | GO:0002702 positive regulation of production of molecular mediator of immune response | 0.014288799 |
| Biological Process | GO:0002011 morphogenesis of an epithelial sheet | 0.014349866 |
| Biological Process | GO:0070670 response to interleukin-4 | 0.014349866 |
| Biological Process | GO:0071622 regulation of granulocyte chemotaxis | 0.014349866 |
| Biological Process | GO:0001558 regulation of cell growth | 0.014832499 |
| Biological Process | GO:0048523 negative regulation of cellular process | 0.01523701 |
| Biological Process | GO:0019882 antigen processing and presentation | 0.015260244 |
| Biological Process | GO:0035455 response to interferon-alpha | 0.015260244 |
| Biological Process | GO:0008283 cell proliferation | 0.015577329 |
| Biological Process | GO:0032963 collagen metabolic process | 0.016559804 |
| Biological Process | GO:0007063 regulation of sister chromatid cohesion | 0.016773577 |
| Biological Process | GO:0072593 reactive oxygen species metabolic process | 0.017579602 |
| Biological Process | GO:0030335 positive regulation of cell migration | 0.017763573 |
| Biological Process | GO:2000147 positive regulation of cell motility | 0.017763573 |
| Biological Process | GO:0001776 leukocyte homeostasis | 0.017870777 |
| Biological Process | GO:0002026 regulation of the force of heart contraction | 0.017880864 |
| Biological Process | GO:0002690 positive regulation of leukocyte chemotaxis | 0.017918529 |
| Biological Process | GO:0006974 cellular response to DNA damage stimulus | 0.017940184 |
| Biological Process | GO:0002237 response to molecule of bacterial origin | 0.018073037 |
| Biological Process | GO:0009306 protein secretion | 0.018202891 |
| Biological Process | GO:0001101 response to acid chemical | 0.018362639 |
| Biological Process | GO:0001894 tissue homeostasis | 0.019006228 |
| Biological Process | GO:0007017 microtubule-based process | 0.019184439 |
| Biological Process | GO:0007275 multicellular organism development | 0.019611808 |
| Biological Process | GO:0007346 regulation of mitotic cell cycle | 0.019611808 |
| Biological Process | GO:1903708 positive regulation of hemopoiesis | 0.019778972 |
| Biological Process | GO:0022616 DNA strand elongation | 0.021588355 |
| Biological Process | GO:0090329 regulation of DNA-dependent DNA replication | 0.021968876 |
| Biological Process | GO:0003283 atrial septum development | 0.02239636 |
| Biological Process | GO:0006333 chromatin assembly or disassembly | 0.023080669 |
| Biological Process | GO:0042102 positive regulation of T cell proliferation | 0.023124848 |
| Biological Process | GO:0050671 positive regulation of lymphocyte proliferation | 0.023124848 |
| Biological Process | GO:0032103 positive regulation of response to external stimulus | 0.025583909 |
| Biological Process | GO:0000018 regulation of DNA recombination | 0.02627883 |
| Biological Process | GO:0031399 regulation of protein modification process | 0.02627883 |
| Biological Process | GO:0051272 positive regulation of cellular component movement | 0.02627883 |
| Biological Process | GO:0045582 positive regulation of T cell differentiation | 0.02683499 |
| Biological Process | GO:0045621 positive regulation of lymphocyte differentiation | 0.02683499 |
| Biological Process | GO:0031294 lymphocyte costimulation | 0.027384301 |
| Biological Process | GO:0043368 positive T cell selection | 0.028096976 |
| Biological Process | GO:0010646 regulation of cell communication | 0.028565289 |
| Biological Process | GO:0023051 regulation of signaling | 0.028565289 |
| Biological Process | GO:0051259 protein oligomerization | 0.028565289 |
| Biological Process | GO:0009966 regulation of signal transduction | 0.028911203 |
| Biological Process | GO:0022403 cell cycle phase | 0.028911203 |
| Biological Process | GO:0042060 wound healing | 0.028911203 |
| Biological Process | GO:0051336 regulation of hydrolase activity | 0.028928048 |
| Biological Process | GO:1903320 regulation of protein modification by small protein conjugation or removal | 0.029146595 |
| Biological Process | GO:0070233 negative regulation of T cell apoptotic process | 0.031272413 |
| Biological Process | GO:0002639 positive regulation of immunoglobulin production | 0.031547245 |
| Biological Process | GO:0002889 regulation of immunoglobulin mediated immune response | 0.031547245 |
| Biological Process | GO:0042308 negative regulation of protein import into nucleus | 0.031547245 |
| Biological Process | GO:0090317 negative regulation of intracellular protein transport | 0.031547245 |
| Biological Process | GO:1900181 negative regulation of protein localization to nucleus | 0.031547245 |
| Biological Process | GO:1903828 negative regulation of cellular protein localization | 0.031547245 |
| Biological Process | GO:1904590 negative regulation of protein import | 0.031547245 |
| Biological Process | GO:0097485 neuron projection guidance | 0.031767685 |
| Biological Process | GO:0051098 regulation of binding | 0.032043612 |
| Biological Process | GO:0071345 cellular response to cytokine stimulus | 0.033171672 |
| Biological Process | GO:0044848 biological phase | 0.034095495 |
| Biological Process | GO:0000724 double-strand break repair via homologous recombination | 0.034132268 |
| Biological Process | GO:0030854 positive regulation of granulocyte differentiation | 0.034132268 |
| Biological Process | GO:0043278 response to morphine | 0.034132268 |
| Biological Process | GO:0044699 single-organism process | 0.034132268 |
| Biological Process | GO:0061209 cell proliferation involved in mesonephros development | 0.034132268 |
| Biological Process | GO:0002456 T cell mediated immunity | 0.034948229 |
| Biological Process | GO:0006461 protein complex assembly | 0.034948229 |
| Biological Process | GO:0006959 humoral immune response | 0.034948229 |
| Biological Process | GO:0032435 negative regulation of proteasomal ubiquitin-dependent protein catabolic process | 0.034948229 |
| Biological Process | GO:0032655 regulation of interleukin-12 production | 0.034948229 |
| Biological Process | GO:0042177 negative regulation of protein catabolic process | 0.034948229 |
| Biological Process | GO:0048522 positive regulation of cellular process | 0.034948229 |
| Biological Process | GO:0050688 regulation of defense response to virus | 0.034948229 |
| Biological Process | GO:0070271 protein complex biogenesis | 0.034948229 |
| Biological Process | GO:1901799 negative regulation of proteasomal protein catabolic process | 0.034948229 |
| Biological Process | GO:1903051 negative regulation of proteolysis involved in cellular protein catabolic process | 0.034948229 |
| Biological Process | GO:1903363 negative regulation of cellular protein catabolic process | 0.034948229 |
| Biological Process | GO:0031401 positive regulation of protein modification process | 0.035722667 |
| Biological Process | GO:0001910 regulation of leukocyte mediated cytotoxicity | 0.035811035 |
| Biological Process | GO:0032651 regulation of interleukin-1 beta production | 0.036105684 |
| Biological Process | GO:0051247 positive regulation of protein metabolic process | 0.0374319 |
| Biological Process | GO:0033554 cellular response to stress | 0.039502088 |
| Biological Process | GO:0042312 regulation of vasodilation | 0.039883067 |
| Biological Process | GO:0043044 ATP-dependent chromatin remodeling | 0.039883067 |
| Biological Process | GO:0043486 histone exchange | 0.039883067 |
| Biological Process | GO:0031497 chromatin assembly | 0.040609469 |
| Biological Process | GO:0002320 lymphoid progenitor cell differentiation | 0.040650808 |
| Biological Process | GO:0016569 covalent chromatin modification | 0.040650808 |
| Biological Process | GO:0016570 histone modification | 0.040650808 |
| Biological Process | GO:0032386 regulation of intracellular transport | 0.040650808 |
| Biological Process | GO:0033045 regulation of sister chromatid segregation | 0.040650808 |
| Biological Process | GO:0042992 negative regulation of transcription factor import into nucleus | 0.040650808 |
| Biological Process | GO:0044093 positive regulation of molecular function | 0.0415485 |
| Biological Process | GO:0044767 single-organism developmental process | 0.041807687 |
| Biological Process | GO:0006956 complement activation | 0.042030948 |
| Biological Process | GO:0007052 mitotic spindle organization | 0.042030948 |
| Biological Process | GO:0001816 cytokine production | 0.042061909 |
| Biological Process | GO:0007264 small GTPase mediated signal transduction | 0.042483063 |
| Biological Process | GO:0002688 regulation of leukocyte chemotaxis | 0.042615325 |
| Biological Process | GO:0008284 positive regulation of cell proliferation | 0.044267832 |
| Biological Process | GO:0016192 vesicle-mediated transport | 0.044991746 |
| Biological Process | GO:0001912 positive regulation of leukocyte mediated cytotoxicity | 0.045097843 |
| Biological Process | GO:0045596 negative regulation of cell differentiation | 0.045097843 |
| Biological Process | GO:0032270 positive regulation of cellular protein metabolic process | 0.045299611 |
| Biological Process | GO:0002683 negative regulation of immune system process | 0.045375535 |
| Biological Process | GO:0002687 positive regulation of leukocyte migration | 0.047455827 |
| Biological Process | GO:0002718 regulation of cytokine production involved in immune response | 0.048540605 |
| Biological Process | GO:0002833 positive regulation of response to biotic stimulus | 0.048540605 |
| Biological Process | GO:0002891 positive regulation of immunoglobulin mediated immune response | 0.048540605 |
| Biological Process | GO:0019724 B cell mediated immunity | 0.048540605 |
| Biological Process | GO:0051224 negative regulation of protein transport | 0.048540605 |
| Biological Process | GO:1904950 negative regulation of establishment of protein localization | 0.048540605 |
| Biological Process | GO:0050727 regulation of inflammatory response | 0.049705478 |
| Cellular Component | GO:0005694 chromosome | 1.25E-16 |
| Cellular Component | GO:0044427 chromosomal part | 1.95E-15 |
| Cellular Component | GO:0043228 non-membrane-bounded organelle | 4.46E-13 |
| Cellular Component | GO:0043232 intracellular non-membrane-bounded organelle | 4.46E-13 |
| Cellular Component | GO:0000785 chromatin | 8.43E-10 |
| Cellular Component | GO:0044459 plasma membrane part | 8.43E-10 |
| Cellular Component | GO:0098687 chromosomal region | 8.48E-09 |
| Cellular Component | GO:0000775 chromosome, centromeric region | 1.43E-08 |
| Cellular Component | GO:0005886 plasma membrane | 1.58E-07 |
| Cellular Component | GO:0071944 cell periphery | 2.30E-07 |
| Cellular Component | GO:0005583 fibrillar collagen trimer | 4.21E-06 |
| Cellular Component | GO:0098643 banded collagen fibril | 4.21E-06 |
| Cellular Component | GO:0098644 complex of collagen trimers | 4.21E-06 |
| Cellular Component | GO:0000790 nuclear chromatin | 5.29E-06 |
| Cellular Component | GO:0000779 condensed chromosome, centromeric region | 3.09E-05 |
| Cellular Component | GO:0000228 nuclear chromosome | 3.42E-05 |
| Cellular Component | GO:0000776 kinetochore | 3.82E-05 |
| Cellular Component | GO:0000793 condensed chromosome | 3.84E-05 |
| Cellular Component | GO:0044454 nuclear chromosome part | 4.21E-05 |
| Cellular Component | GO:0042101 T cell receptor complex | 7.14E-05 |
| Cellular Component | GO:0005856 cytoskeleton | 7.92E-05 |
| Cellular Component | GO:0098797 plasma membrane protein complex | 0.000114275 |
| Cellular Component | GO:0000777 condensed chromosome kinetochore | 0.000522479 |
| Cellular Component | GO:0005623 cell | 0.000997583 |
| Cellular Component | GO:0005819 spindle | 0.000997583 |
| Cellular Component | GO:0044464 cell part | 0.000997583 |
| Cellular Component | GO:0015630 microtubule cytoskeleton | 0.001030135 |
| Cellular Component | GO:0000786 nucleosome | 0.001221085 |
| Cellular Component | GO:0044815 DNA packaging complex | 0.001221085 |
| Cellular Component | GO:0043235 receptor complex | 0.001786226 |
| Cellular Component | GO:0005581 collagen trimer | 0.003160593 |
| Cellular Component | GO:0005578 proteinaceous extracellular matrix | 0.003335088 |
| Cellular Component | GO:0000940 condensed chromosome outer kinetochore | 0.007666302 |
| Cellular Component | GO:0098802 plasma membrane receptor complex | 0.007666302 |
| Cellular Component | GO:0042611 MHC protein complex | 0.015151713 |
| Cellular Component | GO:0030054 cell junction | 0.015307058 |
| Cellular Component | GO:0005874 microtubule | 0.02009253 |
| Cellular Component | GO:0008305 integrin complex | 0.02009253 |
| Cellular Component | GO:0044430 cytoskeletal part | 0.02009253 |
| Cellular Component | GO:0098636 protein complex involved in cell adhesion | 0.02009253 |
| Cellular Component | GO:0005634 nucleus | 0.030899346 |
| Cellular Component | GO:0032993 protein-DNA complex | 0.032791958 |
| Cellular Component | GO:0000323 lytic vacuole | 0.034899334 |
| Cellular Component | GO:0005764 lysosome | 0.047228175 |
| Cellular Component | GO:0031988 membrane-bounded vesicle | 0.047228175 |
| Cellular Component | GO:0044420 extracellular matrix component | 0.047228175 |
| Molecular Function | GO:0005515 protein binding | 1.06E-17 |
| Molecular Function | GO:0005488 binding | 8.36E-09 |
| Molecular Function | GO:0046983 protein dimerization activity | 1.76E-05 |
| Molecular Function | GO:0003823 antigen binding | 0.000124016 |
| Molecular Function | GO:0019899 enzyme binding | 0.000124016 |
| Molecular Function | GO:0019900 kinase binding | 0.000124016 |
| Molecular Function | GO:0003682 chromatin binding | 0.000191446 |
| Molecular Function | GO:0023023 MHC protein complex binding | 0.003780388 |
| Molecular Function | GO:0044877 macromolecular complex binding | 0.009584583 |
| Molecular Function | GO:0043566 structure-specific DNA binding | 0.018650187 |
| Molecular Function | GO:1901363 heterocyclic compound binding | 0.018650187 |
| Molecular Function | GO:0097159 organic cyclic compound binding | 0.023232123 |
| Molecular Function | GO:0004950 chemokine receptor activity | 0.039181876 |
| Molecular Function | GO:0005088 Ras guanyl-nucleotide exchange factor activity | 0.039181876 |
| Molecular Function | GO:0003676 nucleic acid binding | 0.042731182 |
| Molecular Function | GO:0005342 organic acid transmembrane transporter activity | 0.042731182 |
