# Supplementary material for: Transcriptome analysis reveals potential immune function-related regulatory genes/pathways of female Lubo goat submandibular glands at different developmental stages
Source: PeerJ. 2020 Oct 7;8:e9947. doi: 10.7717/peerj.9947 (PMC7547598; doi:10.7717/peerj.9947)
Supplement: Table S8 [file peerj-08-9947-s008.docx]

**Table S9:**

**Significantly enriched GO terms of DEGs between group A and C**

| Type | Term | FDR |
| --- | --- | --- |
| Biological Process | GO:0002376 immune system process | 1.44E-27 |
| Biological Process | GO:0045321 leukocyte activation | 2.78E-25 |
| Biological Process | GO:0046649 lymphocyte activation | 5.51E-24 |
| Biological Process | GO:0001775 cell activation | 1.16E-23 |
| Biological Process | GO:0016337 single organismal cell-cell adhesion | 1.09E-17 |
| Biological Process | GO:0070486 leukocyte aggregation | 1.33E-17 |
| Biological Process | GO:0034109 homotypic cell-cell adhesion | 1.50E-17 |
| Biological Process | GO:0007159 leukocyte cell-cell adhesion | 1.60E-17 |
| Biological Process | GO:0042110 T cell activation | 3.06E-15 |
| Biological Process | GO:0070489 T cell aggregation | 3.06E-15 |
| Biological Process | GO:0071593 lymphocyte aggregation | 3.06E-15 |
| Biological Process | GO:0002682 regulation of immune system process | 2.15E-14 |
| Biological Process | GO:0098609 cell-cell adhesion | 1.74E-12 |
| Biological Process | GO:0030098 lymphocyte differentiation | 4.28E-12 |
| Biological Process | GO:0098602 single organism cell adhesion | 1.15E-11 |
| Biological Process | GO:0007049 cell cycle | 4.25E-11 |
| Biological Process | GO:0007155 cell adhesion | 5.22E-11 |
| Biological Process | GO:0022610 biological adhesion | 7.58E-11 |
| Biological Process | GO:0045619 regulation of lymphocyte differentiation | 3.58E-10 |
| Biological Process | GO:0030217 T cell differentiation | 2.28E-09 |
| Biological Process | GO:0002520 immune system development | 2.63E-09 |
| Biological Process | GO:0050776 regulation of immune response | 2.88E-09 |
| Biological Process | GO:0009605 response to external stimulus | 5.89E-09 |
| Biological Process | GO:0002521 leukocyte differentiation | 1.15E-08 |
| Biological Process | GO:0050851 antigen receptor-mediated signaling pathway | 1.15E-08 |
| Biological Process | GO:0022402 cell cycle process | 1.37E-08 |
| Biological Process | GO:1902105 regulation of leukocyte differentiation | 1.38E-08 |
| Biological Process | GO:1903706 regulation of hemopoiesis | 2.11E-08 |
| Biological Process | GO:0043207 response to external biotic stimulus | 5.34E-08 |
| Biological Process | GO:0016043 cellular component organization | 5.49E-08 |
| Biological Process | GO:0034110 regulation of homotypic cell-cell adhesion | 5.88E-08 |
| Biological Process | GO:0048534 hematopoietic or lymphoid organ development | 5.88E-08 |
| Biological Process | GO:0050854 regulation of antigen receptor-mediated signaling pathway | 5.88E-08 |
| Biological Process | GO:0002684 positive regulation of immune system process | 6.68E-08 |
| Biological Process | GO:0006996 organelle organization | 6.90E-08 |
| Biological Process | GO:0051249 regulation of lymphocyte activation | 7.03E-08 |
| Biological Process | GO:0034728 nucleosome organization | 9.54E-08 |
| Biological Process | GO:0009607 response to biotic stimulus | 1.22E-07 |
| Biological Process | GO:0071840 cellular component organization or biogenesis | 1.22E-07 |
| Biological Process | GO:0006259 DNA metabolic process | 1.22E-07 |
| Biological Process | GO:0051276 chromosome organization | 1.52E-07 |
| Biological Process | GO:0051707 response to other organism | 1.59E-07 |
| Biological Process | GO:0000280 nuclear division | 1.91E-07 |
| Biological Process | GO:0002694 regulation of leukocyte activation | 2.62E-07 |
| Biological Process | GO:0050865 regulation of cell activation | 2.64E-07 |
| Biological Process | GO:0071824 protein-DNA complex subunit organization | 2.74E-07 |
| Biological Process | GO:0006950 response to stress | 3.62E-07 |
| Biological Process | GO:0048285 organelle fission | 5.03E-07 |
| Biological Process | GO:0045580 regulation of T cell differentiation | 7.61E-07 |
| Biological Process | GO:0002252 immune effector process | 8.96E-07 |
| Biological Process | GO:0051262 protein tetramerization | 1.05E-06 |
| Biological Process | GO:0009617 response to bacterium | 1.42E-06 |
| Biological Process | GO:0030097 hemopoiesis | 2.05E-06 |
| Biological Process | GO:0022407 regulation of cell-cell adhesion | 2.31E-06 |
| Biological Process | GO:0050778 positive regulation of immune response | 2.40E-06 |
| Biological Process | GO:0051240 positive regulation of multicellular organismal process | 2.95E-06 |
| Biological Process | GO:0002429 immune response-activating cell surface receptor signaling pathway | 4.47E-06 |
| Biological Process | GO:0042113 B cell activation | 7.81E-06 |
| Biological Process | GO:0071822 protein complex subunit organization | 7.81E-06 |
| Biological Process | GO:0034112 positive regulation of homotypic cell-cell adhesion | 8.00E-06 |
| Biological Process | GO:0001819 positive regulation of cytokine production | 1.11E-05 |
| Biological Process | GO:2000026 regulation of multicellular organismal development | 1.70E-05 |
| Biological Process | GO:0006955 immune response | 1.85E-05 |
| Biological Process | GO:0051239 regulation of multicellular organismal process | 1.91E-05 |
| Biological Process | GO:0002562 somatic diversification of immune receptors via germline recombination within a single locus | 2.62E-05 |
| Biological Process | GO:0016444 somatic cell DNA recombination | 2.62E-05 |
| Biological Process | GO:0071103 DNA conformation change | 2.80E-05 |
| Biological Process | GO:0070661 leukocyte proliferation | 2.86E-05 |
| Biological Process | GO:0050863 regulation of T cell activation | 3.02E-05 |
| Biological Process | GO:1903037 regulation of leukocyte cell-cell adhesion | 3.02E-05 |
| Biological Process | GO:0032508 DNA duplex unwinding | 3.02E-05 |
| Biological Process | GO:0046637 regulation of alpha-beta T cell differentiation | 3.12E-05 |
| Biological Process | GO:0006310 DNA recombination | 3.28E-05 |
| Biological Process | GO:0050900 leukocyte migration | 3.31E-05 |
| Biological Process | GO:0046631 alpha-beta T cell activation | 4.00E-05 |
| Biological Process | GO:0007051 spindle organization | 4.29E-05 |
| Biological Process | GO:0002253 activation of immune response | 4.50E-05 |
| Biological Process | GO:0001817 regulation of cytokine production | 4.83E-05 |
| Biological Process | GO:0002696 positive regulation of leukocyte activation | 5.88E-05 |
| Biological Process | GO:0048513 animal organ development | 6.00E-05 |
| Biological Process | GO:0050896 response to stimulus | 6.07E-05 |
| Biological Process | GO:0043933 macromolecular complex subunit organization | 6.81E-05 |
| Biological Process | GO:0051251 positive regulation of lymphocyte activation | 7.00E-05 |
| Biological Process | GO:0050867 positive regulation of cell activation | 8.40E-05 |
| Biological Process | GO:0006935 chemotaxis | 8.40E-05 |
| Biological Process | GO:0042330 taxis | 8.40E-05 |
| Biological Process | GO:0006325 chromatin organization | 9.32E-05 |
| Biological Process | GO:0032943 mononuclear cell proliferation | 9.74E-05 |
| Biological Process | GO:0050794 regulation of cellular process | 9.74E-05 |
| Biological Process | GO:0046634 regulation of alpha-beta T cell activation | 0.000126168 |
| Biological Process | GO:0022409 positive regulation of cell-cell adhesion | 0.000142952 |
| Biological Process | GO:0045058 T cell selection | 0.000144483 |
| Biological Process | GO:0002768 immune response-regulating cell surface receptor signaling pathway | 0.000154546 |
| Biological Process | GO:0048583 regulation of response to stimulus | 0.000164687 |
| Biological Process | GO:0050789 regulation of biological process | 0.000171697 |
| Biological Process | GO:0002200 somatic diversification of immune receptors | 0.000171697 |
| Biological Process | GO:0046651 lymphocyte proliferation | 0.00019684 |
| Biological Process | GO:0002757 immune response-activating signal transduction | 0.000220701 |
| Biological Process | GO:0032743 positive regulation of interleukin-2 production | 0.000279249 |
| Biological Process | GO:0040011 locomotion | 0.000280069 |
| Biological Process | GO:0034502 protein localization to chromosome | 0.000295987 |
| Biological Process | GO:0050855 regulation of B cell receptor signaling pathway | 0.00029985 |
| Biological Process | GO:0048584 positive regulation of response to stimulus | 0.000359541 |
| Biological Process | GO:0002443 leukocyte mediated immunity | 0.00038853 |
| Biological Process | GO:0030155 regulation of cell adhesion | 0.00038853 |
| Biological Process | GO:0050870 positive regulation of T cell activation | 0.00038853 |
| Biological Process | GO:1903039 positive regulation of leukocyte cell-cell adhesion | 0.00038853 |
| Biological Process | GO:0006928 movement of cell or subcellular component | 0.000402168 |
| Biological Process | GO:0051726 regulation of cell cycle | 0.000402168 |
| Biological Process | GO:0032663 regulation of interleukin-2 production | 0.000477735 |
| Biological Process | GO:0030595 leukocyte chemotaxis | 0.000594094 |
| Biological Process | GO:0000278 mitotic cell cycle | 0.000616776 |
| Biological Process | GO:1903650 negative regulation of cytoplasmic transport | 0.000621039 |
| Biological Process | GO:0065007 biological regulation | 0.000871128 |
| Biological Process | GO:0032387 negative regulation of intracellular transport | 0.001005522 |
| Biological Process | GO:0002263 cell activation involved in immune response | 0.001080099 |
| Biological Process | GO:0002366 leukocyte activation involved in immune response | 0.001080099 |
| Biological Process | GO:0043370 regulation of CD4-positive, alpha-beta T cell differentiation | 0.001090406 |
| Biological Process | GO:2000514 regulation of CD4-positive, alpha-beta T cell activation | 0.001090406 |
| Biological Process | GO:0044707 single-multicellular organism process | 0.001175873 |
| Biological Process | GO:2000106 regulation of leukocyte apoptotic process | 0.001232129 |
| Biological Process | GO:0036037 CD8-positive, alpha-beta T cell activation | 0.001294725 |
| Biological Process | GO:0060326 cell chemotaxis | 0.001327411 |
| Biological Process | GO:0051983 regulation of chromosome segregation | 0.001332542 |
| Biological Process | GO:0050793 regulation of developmental process | 0.001340137 |
| Biological Process | GO:0061640 cytoskeleton-dependent cytokinesis | 0.001363685 |
| Biological Process | GO:0035710 CD4-positive, alpha-beta T cell activation | 0.001437889 |
| Biological Process | GO:0002764 immune response-regulating signaling pathway | 0.001484544 |
| Biological Process | GO:0003230 cardiac atrium development | 0.001484544 |
| Biological Process | GO:0045785 positive regulation of cell adhesion | 0.001511799 |
| Biological Process | GO:1903047 mitotic cell cycle process | 0.001549093 |
| Biological Process | GO:0070229 negative regulation of lymphocyte apoptotic process | 0.001653529 |
| Biological Process | GO:2000107 negative regulation of leukocyte apoptotic process | 0.001653529 |
| Biological Process | GO:0031577 spindle checkpoint | 0.001653529 |
| Biological Process | GO:0071621 granulocyte chemotaxis | 0.001653529 |
| Biological Process | GO:0048731 system development | 0.001891724 |
| Biological Process | GO:0006952 defense response | 0.001978381 |
| Biological Process | GO:0009620 response to fungus | 0.001978963 |
| Biological Process | GO:0010564 regulation of cell cycle process | 0.002073802 |
| Biological Process | GO:0097530 granulocyte migration | 0.002301607 |
| Biological Process | GO:0006323 DNA packaging | 0.002301607 |
| Biological Process | GO:0007275 multicellular organism development | 0.002508049 |
| Biological Process | GO:0042098 T cell proliferation | 0.002666169 |
| Biological Process | GO:0006897 endocytosis | 0.00283209 |
| Biological Process | GO:0042742 defense response to bacterium | 0.00283209 |
| Biological Process | GO:0051259 protein oligomerization | 0.00299656 |
| Biological Process | GO:0002705 positive regulation of leukocyte mediated immunity | 0.003031114 |
| Biological Process | GO:0002274 myeloid leukocyte activation | 0.003176208 |
| Biological Process | GO:0000212 meiotic spindle organization | 0.003242406 |
| Biological Process | GO:0032875 regulation of DNA endoreduplication | 0.003242406 |
| Biological Process | GO:0006305 DNA alkylation | 0.003408836 |
| Biological Process | GO:0000075 cell cycle checkpoint | 0.003409714 |
| Biological Process | GO:0001776 leukocyte homeostasis | 0.003409714 |
| Biological Process | GO:0030154 cell differentiation | 0.003469856 |
| Biological Process | GO:0002449 lymphocyte mediated immunity | 0.00349964 |
| Biological Process | GO:0097529 myeloid leukocyte migration | 0.003533199 |
| Biological Process | GO:0048519 negative regulation of biological process | 0.003585387 |
| Biological Process | GO:0048870 cell motility | 0.003673139 |
| Biological Process | GO:0051674 localization of cell | 0.003673139 |
| Biological Process | GO:0000226 microtubule cytoskeleton organization | 0.004038505 |
| Biological Process | GO:0098542 defense response to other organism | 0.004301458 |
| Biological Process | GO:0045582 positive regulation of T cell differentiation | 0.004301458 |
| Biological Process | GO:0045621 positive regulation of lymphocyte differentiation | 0.004301458 |
| Biological Process | GO:0050670 regulation of lymphocyte proliferation | 0.004838316 |
| Biological Process | GO:0031347 regulation of defense response | 0.004900869 |
| Biological Process | GO:0043383 negative T cell selection | 0.005272009 |
| Biological Process | GO:0001818 negative regulation of cytokine production | 0.005384887 |
| Biological Process | GO:0044763 single-organism cellular process | 0.005546938 |
| Biological Process | GO:0002819 regulation of adaptive immune response | 0.005956272 |
| Biological Process | GO:0046823 negative regulation of nucleocytoplasmic transport | 0.006085801 |
| Biological Process | GO:0048518 positive regulation of biological process | 0.006739665 |
| Biological Process | GO:0031348 negative regulation of defense response | 0.006739665 |
| Biological Process | GO:0032651 regulation of interleukin-1 beta production | 0.006981243 |
| Biological Process | GO:0001906 cell killing | 0.007004479 |
| Biological Process | GO:1902589 single-organism organelle organization | 0.007103905 |
| Biological Process | GO:0002697 regulation of immune effector process | 0.007492125 |
| Biological Process | GO:0002821 positive regulation of adaptive immune response | 0.008308387 |
| Biological Process | GO:0002824 positive regulation of adaptive immune response based on somatic recombination of immune receptors built from immunoglobulin superfamily domains | 0.008308387 |
| Biological Process | GO:0045595 regulation of cell differentiation | 0.008308387 |
| Biological Process | GO:0060413 atrial septum morphogenesis | 0.008804906 |
| Biological Process | GO:0050864 regulation of B cell activation | 0.008851449 |
| Biological Process | GO:0032944 regulation of mononuclear cell proliferation | 0.009079033 |
| Biological Process | GO:0032844 regulation of homeostatic process | 0.00911076 |
| Biological Process | GO:0006954 inflammatory response | 0.009452287 |
| Biological Process | GO:0042129 regulation of T cell proliferation | 0.009914632 |
| Biological Process | GO:0009611 response to wounding | 0.009914632 |
| Biological Process | GO:0071379 cellular response to prostaglandin stimulus | 0.009914632 |
| Biological Process | GO:0071398 cellular response to fatty acid | 0.009914632 |
| Biological Process | GO:0002714 positive regulation of B cell mediated immunity | 0.010271943 |
| Biological Process | GO:0032434 regulation of proteasomal ubiquitin-dependent protein catabolic process | 0.010271943 |
| Biological Process | GO:0045830 positive regulation of isotype switching | 0.010271943 |
| Biological Process | GO:0045911 positive regulation of DNA recombination | 0.010271943 |
| Biological Process | GO:0050871 positive regulation of B cell activation | 0.010271943 |
| Biological Process | GO:0002703 regulation of leukocyte mediated immunity | 0.010568258 |
| Biological Process | GO:0033554 cellular response to stress | 0.010752632 |
| Biological Process | GO:0050856 regulation of T cell receptor signaling pathway | 0.011293634 |
| Biological Process | GO:0007059 chromosome segregation | 0.012032793 |
| Biological Process | GO:0034097 response to cytokine | 0.01212574 |
| Biological Process | GO:0070228 regulation of lymphocyte apoptotic process | 0.012316668 |
| Biological Process | GO:1903708 positive regulation of hemopoiesis | 0.012319002 |
| Biological Process | GO:0008356 asymmetric cell division | 0.01349715 |
| Biological Process | GO:0042415 norepinephrine metabolic process | 0.01349715 |
| Biological Process | GO:0002699 positive regulation of immune effector process | 0.013519306 |
| Biological Process | GO:0002706 regulation of lymphocyte mediated immunity | 0.013773397 |
| Biological Process | GO:0007063 regulation of sister chromatid cohesion | 0.013773397 |
| Biological Process | GO:0033599 regulation of mammary gland epithelial cell proliferation | 0.013773397 |
| Biological Process | GO:0002250 adaptive immune response | 0.013861376 |
| Biological Process | GO:0002460 adaptive immune response based on somatic recombination of immune receptors built from immunoglobulin superfamily domains | 0.013861376 |
| Biological Process | GO:0070663 regulation of leukocyte proliferation | 0.014364687 |
| Biological Process | GO:0051054 positive regulation of DNA metabolic process | 0.01462919 |
| Biological Process | GO:0001101 response to acid chemical | 0.015595392 |
| Biological Process | GO:0065009 regulation of molecular function | 0.015598203 |
| Biological Process | GO:0007010 cytoskeleton organization | 0.016524592 |
| Biological Process | GO:0090329 regulation of DNA-dependent DNA replication | 0.017477999 |
| Biological Process | GO:0042102 positive regulation of T cell proliferation | 0.01749415 |
| Biological Process | GO:0050671 positive regulation of lymphocyte proliferation | 0.01749415 |
| Biological Process | GO:0002822 regulation of adaptive immune response based on somatic recombination of immune receptors built from immunoglobulin superfamily domains | 0.017884054 |
| Biological Process | GO:0002708 positive regulation of lymphocyte mediated immunity | 0.017884054 |
| Biological Process | GO:0000018 regulation of DNA recombination | 0.017884054 |
| Biological Process | GO:0022403 cell cycle phase | 0.017884054 |
| Biological Process | GO:0032392 DNA geometric change | 0.018716981 |
| Biological Process | GO:0048523 negative regulation of cellular process | 0.019431918 |
| Biological Process | GO:0003283 atrial septum development | 0.019431918 |
| Biological Process | GO:0060249 anatomical structure homeostasis | 0.019431918 |
| Biological Process | GO:0030198 extracellular matrix organization | 0.019545926 |
| Biological Process | GO:0022616 DNA strand elongation | 0.019670658 |
| Biological Process | GO:0036072 direct ossification | 0.019670658 |
| Biological Process | GO:0032652 regulation of interleukin-1 production | 0.020202233 |
| Biological Process | GO:0002712 regulation of B cell mediated immunity | 0.020896663 |
| Biological Process | GO:2000021 regulation of ion homeostasis | 0.020913986 |
| Biological Process | GO:0044848 biological phase | 0.021234529 |
| Biological Process | GO:0030335 positive regulation of cell migration | 0.023320792 |
| Biological Process | GO:2000147 positive regulation of cell motility | 0.023320792 |
| Biological Process | GO:0008277 regulation of G-protein coupled receptor protein signaling pathway | 0.023575641 |
| Biological Process | GO:0051052 regulation of DNA metabolic process | 0.024711096 |
| Biological Process | GO:0001894 tissue homeostasis | 0.025154621 |
| Biological Process | GO:0002639 positive regulation of immunoglobulin production | 0.025154621 |
| Biological Process | GO:0003209 cardiac atrium morphogenesis | 0.027296524 |
| Biological Process | GO:0070233 negative regulation of T cell apoptotic process | 0.027296524 |
| Biological Process | GO:0007264 small GTPase mediated signal transduction | 0.027887041 |
| Biological Process | GO:0010033 response to organic substance | 0.028305287 |
| Biological Process | GO:0010522 regulation of calcium ion transport into cytosol | 0.028416203 |
| Biological Process | GO:0040017 positive regulation of locomotion | 0.028475884 |
| Biological Process | GO:0045191 regulation of isotype switching | 0.02883049 |
| Biological Process | GO:0045596 negative regulation of cell differentiation | 0.028925579 |
| Biological Process | GO:0070887 cellular response to chemical stimulus | 0.029363678 |
| Biological Process | GO:0033043 regulation of organelle organization | 0.029842077 |
| Biological Process | GO:0035556 intracellular signal transduction | 0.029950157 |
| Biological Process | GO:0002440 production of molecular mediator of immune response | 0.030611104 |
| Biological Process | GO:0031497 chromatin assembly | 0.030611104 |
| Biological Process | GO:0002637 regulation of immunoglobulin production | 0.03167288 |
| Biological Process | GO:0002685 regulation of leukocyte migration | 0.032012607 |
| Biological Process | GO:1903649 regulation of cytoplasmic transport | 0.032666215 |
| Biological Process | GO:0006974 cellular response to DNA damage stimulus | 0.032666215 |
| Biological Process | GO:0051272 positive regulation of cellular component movement | 0.032666215 |
| Biological Process | GO:0002456 T cell mediated immunity | 0.032666215 |
| Biological Process | GO:0032435 negative regulation of proteasomal ubiquitin-dependent protein catabolic process | 0.032666215 |
| Biological Process | GO:0032655 regulation of interleukin-12 production | 0.032666215 |
| Biological Process | GO:0042177 negative regulation of protein catabolic process | 0.032666215 |
| Biological Process | GO:1901799 negative regulation of proteasomal protein catabolic process | 0.032666215 |
| Biological Process | GO:1903051 negative regulation of proteolysis involved in cellular protein catabolic process | 0.032666215 |
| Biological Process | GO:1903363 negative regulation of cellular protein catabolic process | 0.032666215 |
| Biological Process | GO:0000724 double-strand break repair via homologous recombination | 0.032706481 |
| Biological Process | GO:0043278 response to morphine | 0.032706481 |
| Biological Process | GO:0061209 cell proliferation involved in mesonephros development | 0.032706481 |
| Biological Process | GO:0051319 G2 phase | 0.032706481 |
| Biological Process | GO:0006304 DNA modification | 0.033093604 |
| Biological Process | GO:0002690 positive regulation of leukocyte chemotaxis | 0.033254499 |
| Biological Process | GO:0007017 microtubule-based process | 0.033254499 |
| Biological Process | GO:0033045 regulation of sister chromatid segregation | 0.033254499 |
| Biological Process | GO:0042992 negative regulation of transcription factor import into nucleus | 0.033254499 |
| Biological Process | GO:0007052 mitotic spindle organization | 0.033254499 |
| Biological Process | GO:0034694 response to prostaglandin | 0.033254499 |
| Biological Process | GO:0051301 cell division | 0.033254499 |
| Biological Process | GO:0071624 positive regulation of granulocyte chemotaxis | 0.033291675 |
| Biological Process | GO:0048869 cellular developmental process | 0.033291675 |
| Biological Process | GO:0071345 cellular response to cytokine stimulus | 0.033291675 |
| Biological Process | GO:0043044 ATP-dependent chromatin remodeling | 0.033291675 |
| Biological Process | GO:0043486 histone exchange | 0.033291675 |
| Biological Process | GO:0032731 positive regulation of interleukin-1 beta production | 0.033291675 |
| Biological Process | GO:0032732 positive regulation of interleukin-1 production | 0.033291675 |
| Biological Process | GO:0002260 lymphocyte homeostasis | 0.033333473 |
| Biological Process | GO:0016477 cell migration | 0.03412206 |
| Biological Process | GO:1902107 positive regulation of leukocyte differentiation | 0.034369496 |
| Biological Process | GO:0002285 lymphocyte activation involved in immune response | 0.035766009 |
| Biological Process | GO:0097485 neuron projection guidance | 0.035766009 |
| Biological Process | GO:0002275 myeloid cell activation involved in immune response | 0.038149989 |
| Biological Process | GO:0016575 histone deacetylation | 0.038149989 |
| Biological Process | GO:0032946 positive regulation of mononuclear cell proliferation | 0.038211868 |
| Biological Process | GO:0006461 protein complex assembly | 0.039289898 |
| Biological Process | GO:0070271 protein complex biogenesis | 0.039289898 |
| Biological Process | GO:0002891 positive regulation of immunoglobulin mediated immune response | 0.039712094 |
| Biological Process | GO:0051098 regulation of binding | 0.043861039 |
| Biological Process | GO:0031343 positive regulation of cell killing | 0.043861039 |
| Biological Process | GO:0050848 regulation of calcium-mediated signaling | 0.045196257 |
| Biological Process | GO:0006333 chromatin assembly or disassembly | 0.045196257 |
| Biological Process | GO:0042127 regulation of cell proliferation | 0.045594429 |
| Biological Process | GO:0035712 T-helper 2 cell activation | 0.04801179 |
| Biological Process | GO:0090288 negative regulation of cellular response to growth factor stimulus | 0.04801179 |
| Biological Process | GO:0070670 response to interleukin-4 | 0.048426416 |
| Cellular Component | GO:0005694 chromosome | 2.61E-17 |
| Cellular Component | GO:0044427 chromosomal part | 5.67E-16 |
| Cellular Component | GO:0098687 chromosomal region | 5.28E-12 |
| Cellular Component | GO:0043228 non-membrane-bounded organelle | 4.33E-11 |
| Cellular Component | GO:0043232 intracellular non-membrane-bounded organelle | 4.33E-11 |
| Cellular Component | GO:0000775 chromosome, centromeric region | 2.77E-08 |
| Cellular Component | GO:0044459 plasma membrane part | 2.08E-07 |
| Cellular Component | GO:0000793 condensed chromosome | 4.17E-07 |
| Cellular Component | GO:0000785 chromatin | 5.00E-07 |
| Cellular Component | GO:0005886 plasma membrane | 5.64E-07 |
| Cellular Component | GO:0000776 kinetochore | 5.64E-07 |
| Cellular Component | GO:0071944 cell periphery | 7.28E-07 |
| Cellular Component | GO:0000779 condensed chromosome, centromeric region | 1.06E-06 |
| Cellular Component | GO:0005583 fibrillar collagen trimer | 1.92E-06 |
| Cellular Component | GO:0098643 banded collagen fibril | 1.92E-06 |
| Cellular Component | GO:0098644 complex of collagen trimers | 1.92E-06 |
| Cellular Component | GO:0000228 nuclear chromosome | 2.69E-05 |
| Cellular Component | GO:0044454 nuclear chromosome part | 4.54E-05 |
| Cellular Component | GO:0042101 T cell receptor complex | 5.23E-05 |
| Cellular Component | GO:0000790 nuclear chromatin | 0.000110101 |
| Cellular Component | GO:0005623 cell | 0.000347538 |
| Cellular Component | GO:0044464 cell part | 0.000347538 |
| Cellular Component | GO:0000777 condensed chromosome kinetochore | 0.000351409 |
| Cellular Component | GO:0005581 collagen trimer | 0.000351409 |
| Cellular Component | GO:0043073 germ cell nucleus | 0.00126581 |
| Cellular Component | GO:0000803 sex chromosome | 0.001626811 |
| Cellular Component | GO:0005856 cytoskeleton | 0.00191548 |
| Cellular Component | GO:0043235 receptor complex | 0.002064493 |
| Cellular Component | GO:0005578 proteinaceous extracellular matrix | 0.00606013 |
| Cellular Component | GO:0000786 nucleosome | 0.006154107 |
| Cellular Component | GO:0044815 DNA packaging complex | 0.006154107 |
| Cellular Component | GO:0000940 condensed chromosome outer kinetochore | 0.006574466 |
| Cellular Component | GO:0098797 plasma membrane protein complex | 0.008987707 |
| Cellular Component | GO:0005819 spindle | 0.011601832 |
| Cellular Component | GO:0044430 cytoskeletal part | 0.012418446 |
| Cellular Component | GO:0098802 plasma membrane receptor complex | 0.013403125 |
| Cellular Component | GO:0043234 protein complex | 0.015722574 |
| Cellular Component | GO:0015630 microtubule cytoskeleton | 0.018182309 |
| Cellular Component | GO:0032993 protein-DNA complex | 0.023493074 |
| Cellular Component | GO:0044420 extracellular matrix component | 0.025834998 |
| Cellular Component | GO:0005874 microtubule | 0.027778041 |
| Molecular Function | GO:0005515 protein binding | 1.16E-12 |
| Molecular Function | GO:0005488 binding | 2.94E-10 |
| Molecular Function | GO:0046983 protein dimerization activity | 0.00015212 |
| Molecular Function | GO:1901363 heterocyclic compound binding | 0.001042009 |
| Molecular Function | GO:0019900 kinase binding | 0.001500577 |
| Molecular Function | GO:0097159 organic cyclic compound binding | 0.001500577 |
| Molecular Function | GO:0003677 DNA binding | 0.005346393 |
| Molecular Function | GO:0004950 chemokine receptor activity | 0.006618909 |
| Molecular Function | GO:0005342 organic acid transmembrane transporter activity | 0.008405276 |
| Molecular Function | GO:0004896 cytokine receptor activity | 0.008405276 |
| Molecular Function | GO:0019899 enzyme binding | 0.0086374 |
| Molecular Function | GO:0003676 nucleic acid binding | 0.01884973 |
| Molecular Function | GO:0001637 G-protein coupled chemoattractant receptor activity | 0.044026092 |
| Molecular Function | GO:0004955 prostaglandin receptor activity | 0.045502008 |
| Molecular Function | GO:0003823 antigen binding | 0.046324179 |
| Molecular Function | GO:0046943 carboxylic acid transmembrane transporter activity | 0.046324179 |
| Molecular Function | GO:0043566 structure-specific DNA binding | 0.046973869 |
| Molecular Function | GO:0042393 histone binding | 0.046973869 |
| Molecular Function | GO:0097367 carbohydrate derivative binding | 0.048396498 |
| Molecular Function | GO:0004953 icosanoid receptor activity | 0.048396498 |
| Molecular Function | GO:0004954 prostanoid receptor activity | 0.048396498 |
